# Computational Cell Cycle Profiling of Cancer Cells for Prioritizing FDA-Approved Drugs with Repurposing Potential

**DOI:** 10.1038/s41598-017-11508-2

**Published:** 2017-09-12

**Authors:** Yu-Chen Lo, Silvia Senese, Bryan France, Ankur A. Gholkar, Robert Damoiseaux, Jorge Z. Torres

**Affiliations:** 1 Department of Chemistry and Biochemistry, University of California, Los Angeles, CA 90095 USA; 2 Program in Bioengineering, University of California, Los Angeles, CA 90095 USA; 3 Department of Molecular and Medical Pharmacology, Los Angeles, CA 90095 USA; 4 California NanoSystems Institute, University of California, Los Angeles, CA 90095 USA; 5 Jonsson Comprehensive Cancer Center, University of California, Los Angeles, CA 90095 USA; 6 Molecular Biology Institute, University of California, Los Angeles, CA 90095 USA

## Abstract

Discovery of first-in-class medicines for treating cancer is limited by concerns with their toxicity and safety profiles, while repurposing known drugs for new anticancer indications has become a viable alternative. Here, we have developed a new approach that utilizes cell cycle arresting patterns as unique molecular signatures for prioritizing FDA-approved drugs with repurposing potential. As proof-of-principle, we conducted large-scale cell cycle profiling of 884 FDA-approved drugs. Using cell cycle indexes that measure changes in cell cycle profile patterns upon chemical perturbation, we identified 36 compounds that inhibited cancer cell viability including 6 compounds that were previously undescribed. Further cell cycle fingerprint analysis and 3D chemical structural similarity clustering identified unexpected FDA-approved drugs that induced DNA damage, including clinically relevant microtubule destabilizers, which was confirmed experimentally *via* cell-based assays. Our study shows that computational cell cycle profiling can be used as an approach for prioritizing FDA-approved drugs with repurposing potential, which could aid the development of cancer therapeutics.

## Introduction

Cancer remains a debilitating disease that affects millions of people in the US and around the world. Despite tremendous investments in cancer drug discovery including high-throughput screening and structure-based drug design, there has not been a significant increase in the number of new anticancer drugs introduced into the clinics^1^. Additionally, the length of time required for developing a new drug has increased from an average of 7.9 years to 13.9 years and the average expenditure to introduce a new drug to the market is ~1.8 billion US$^1, 2^. The high attrition rate of lead anticancer compounds can often be attributed to their lack of efficacy or unwanted toxicities that arise during clinical trials^3^. On the other hand, FDA-approved drugs have acceptable safety profiles and pharmacokinetic properties relating to absorption, metabolism and toxicity. Consequently, identifying known drugs for new antineoplastic indications, known as “drug repurposing”, “drug repositioning” or “therapeutic switching”, represents a promising strategy to accelerate the approval and clinical application of these drugs for the treatment of cancer. It is estimated that drug repurposing could effectively reduce the drug development time down to 3 years by significantly shortening of the lead optimization phase^4^. The basic idea behind drug repurposing is “poly-pharmacology”, which suggests that a drug not only interacts with a primary target, but also with multiple secondary off-targets. Thus, it is possible to repurpose the drug mechanism important for the treatment of the original indication to target other secondary indications. Furthermore, repurposing known drugs for new indications only requires minimal or no structural modifications that enable rapid drug approval and entry into the clinics.

Several approaches for drug repurposing have been proposed^2, 5^. Early repurposed drugs were discovered serendipitously due to their unexpected side effects. One notable example is sildenafil (Viagra), a well-known drug used for the treatment of erectile dysfunction whose initial indication was for the treatment of heart disease^6^. Recent drug repositioning efforts for the discovery of anticancer agents have utilized a myriad of approaches including high-throughput activity-based screens of disease phenotypes as well as *in-silico* prediction algorithms^2, 7–12^. Nonetheless, mechanism-based drug repurposing that relies on the existing knowledge of a protein target or drug activity often does not directly correlate to a high-level of cellular phenotypic effects, due to potential drug off-target interactions. While high-throughput chemical screening remains an effective strategy for drug repositioning, it offers little mechanistic insight on the identified compounds, making it a challenge for hit prioritization and hit-to-lead optimization. Therefore, there is a critical need to develop more effective approaches for prioritizing FDA-approved drugs with repurposing potential that could aid the development of new cancer drugs.

**Figure 1:**
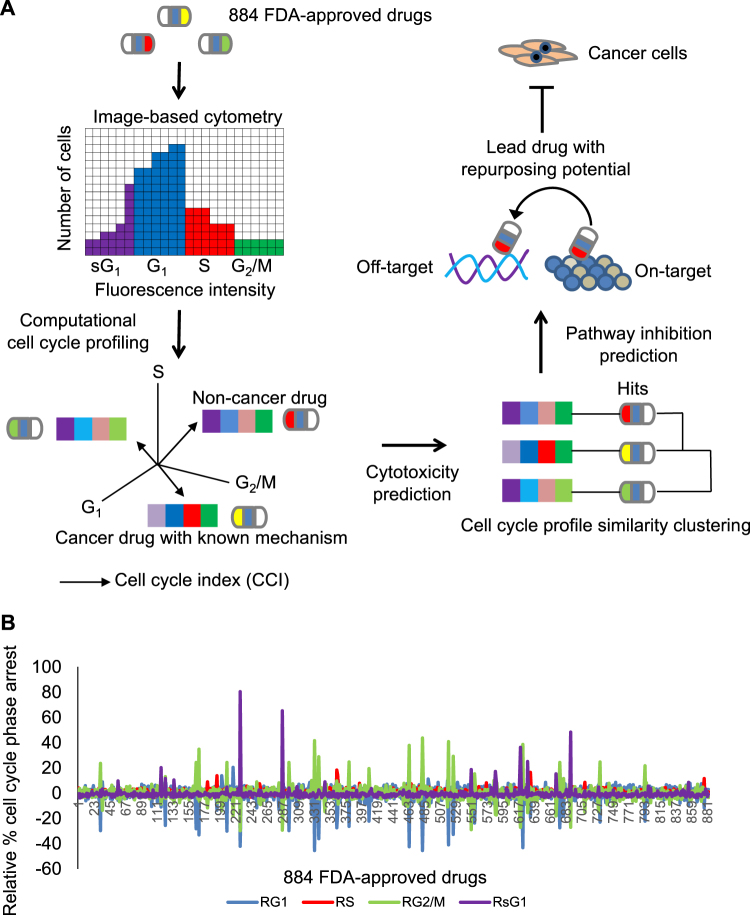
Computational cell cycle profiling for prioritizing FDA-approved drugs with repurposing potential. (**A**) Overview of the computational cell cycle profiling approach for prioritizing FDA-approved drugs with repurposing potential. FDA-approved drugs, with sound pharmacological and safety profiles used to treat broad conditions, are analyzed for their effect on the cell cycle of cancer cells through image-based cytometry. Cell cycle fingerprints are then used to compute a cell cycle index (CCI) that measures the deviation form control cell cycles. The cell cycle fingerprints and CCI are used to computationally predict a drugs cytotoxicity and pathway inhibition. Predictions are further evaluated by experimental cell based assays to define lead drugs with repurposing potential. (**B**) Drug-induced cell cycle profiles of 884 FDA-approved drugs were expressed as cell cycle fingerprints consisting of G_1_, S, G_2_/M and subG_1_ phases relative to the DMSO control profile. The diagram displays the relative percent cell cycle phase arrest on the y-axis for each of the 884 FDA-approved drugs on the x-axis. The four cell cycle phases are color coded; G_1_ (blue), S (red), G_2_/M (green), and subG_1_ (purple). Note that FDA-approved drugs induce a wide variety of cell cycle arrest patterns.

In this study, we report a new approach to prioritize FDA-approved drugs with repurposing potential that utilizes computational cell cycle profiling (Fig. 1A). The progression of cancer relies on the ability of cancer cells to transition through the cell cycle, which consists of G_1_, S, G_2_ and M phases, in order to proliferate^13^. Each cell cycle phase is regulated by cell cycle checkpoints that detect cellular damage and arrest cells to repair damage^14–17^. However, if cellular damage cannot be repaired, cell death pathways like apoptosis are induced to remove the damaged cells^18^. Hence, inhibition of the cell cycle with agents that cause cellular damage during specific phases of the cell cycle has been a viable approach for developing anticancer agents^19^. Although anticancer compounds like staurosporine (G_2_-phase inhibitor), camptothecin (S-phase inhibitor) and paclitaxel (M-phase inhibitor) induce a high percentage of cells to arrest in specific phases of the cell cycle, there are few studies on how the overall cell cycle profile changes in response to these agents^20–22^. Our recent cell cycle profiling of >84,000 drug-like molecules demonstrated that wide variations in cell cycle profiles existed even for compounds arresting cells predominantly in the same phase^19^. This is consistent with substantial *in vivo* and *in vitro* evidence that cancer cells often miss-regulate their cell cycle checkpoints to promote proliferation even under unfavorable external conditions or cellular damage. Similarly, cancer cells display drastic variations *in vitro* in their response to chemotherapeutic agents not only between different cancer cell types but also within the same cancer cell line, which can be partly explained by the functional status of their cell cycle checkpoints^23^. Along with our previous studies, here we highlighted the limitations of current approaches that analyze cell cycle modulators based on a single cell cycle phase and propose a new multi cell cycle phase analysis for prioritizing lead compounds for therapeutic development. In this study, we have established a computational cell cycle profiling approach by considering drug induced changes in G_1_, S, G_2_/M and subG_1_ cell cycle phases to prioritize FDA-approved drugs with repurposing potential. The application of this approach identified 36 FDA-approved drugs that reduced cancer cell viability, including several clinically relevant microtubule destabilizing agents that also elicited DNA damage. These results offer further opportunities to develop new chemotherapies that induce both microtubule and DNA damage.

## Results

**Figure 2:**
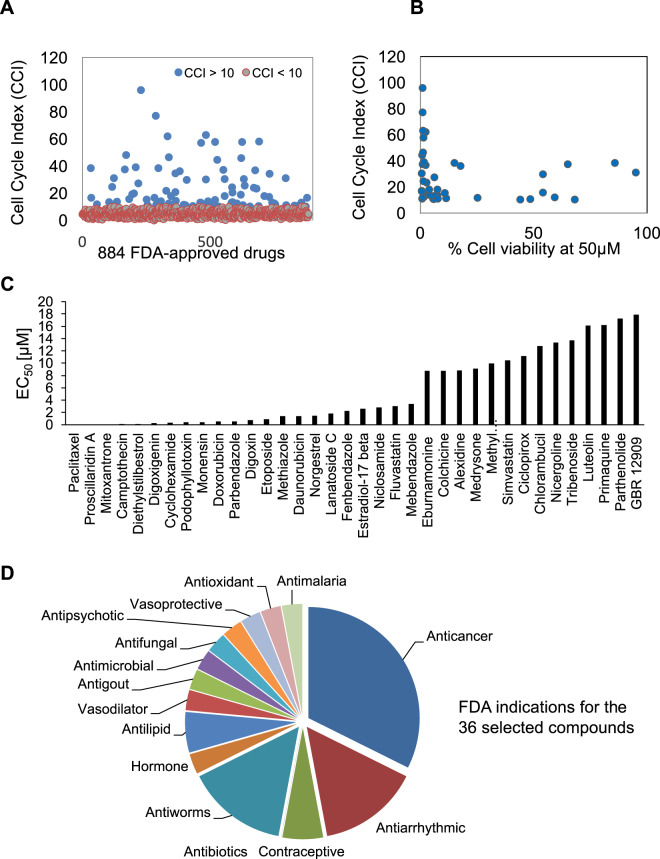
Evaluating drug induced cell cycle profiles and drug effect on cell viability. (**A**) The FDA-approved drug-induced cell cycle profiles were used to compute a cell cycle index (CCI), which measures the Euclidean distance between drug-induced and DMSO control profiles, for each of the 884 FDA-approved drugs. The graph displays the CCI on the y-axis and the 884 drugs on the x-axis. Note that 91 FDA-approved drugs with a CCI > 10 (blue circles), representing those with the strongest cell cycle profile deviations, were selected for further evaluation. For details see Methods. (**B**) The 91 FDA-approved drugs with a CCI > 10 were evaluated for their ability to inhibit HeLa cell viability after a 72 hour treatment at 50 µM final concentration. 46 of these drugs (blue circles) inhibited HeLa cell viability with >3 standard deviations relative to DMSO and were mapped onto their CCI values. Graph displays the percent cell viability on the x-axis and the CCI on the y-axis for each of the 46 drugs represented by blue circles. (**C**) HeLa cells were treated with increasing concentrations of each of the 46 selected FDA-approved drugs for 72 hours and their half maximal effective concentration (EC_50_) was determined. Graph displays the EC_50_ (in µM) for each drug on y-axis and the drug names on the x-axis. Note that 36 drugs displayed EC_50s_ < 20 μM. (**D**) Pie chart summarizing the indications of the 36 most potent FDA-approved drugs that inhibit cancer cell viability. Note that most were not originally indicated for the treatment of cancer.

To test the utility of cell cycle profiling for prioritizing FDA-approved drugs as lead drugs for developing cancer therapeutics, we performed large-scale cell cycle profiling of 884 FDA-approved drugs (Supplementary Table 1). HeLa cancer cells were plated into 384-well plates and a library consisting of 884 FDA-approved drugs was used to place one compound per well at 10 μM final concentration. Twenty hours later the cells were fixed and stained with the DNA-selective stain Vybrant DyeCycle Green, which emits a fluorescent signal after binding to DNA that is proportional to DNA mass when exited at 488 nm^24^. Plates were scanned with a fluorescence microplate cytometer and a cell cycle histogram profile was generated for each well, which had been treated with one FDA compound (Fig. 1A). The control DMSO cell cycle profile indicated that more than 50% of cells were in G_1_ phase, 30% in G_2_/M phase, 10% in S phase and less than 5% in a subG_1_ phase likely due to apoptotic cell death (Supplementary Table 2). In contrast, the known antimitotic drug taxol arrested 80% of the cells in G_2_/M phase (Supplementary Table 2). To further quantify cell cycle phase changes, we converted cell cycle profiles into fingerprints consisting of four cell cycle phases (G_1_, S, G_2_/M and subG_1_) and computed a cell cycle index (CCI) based on the Euclidean distance between drug-treated and DMSO-treated profiles (Fig. 1B). To identify the FDA-approved drugs that induced the strongest deviations in the cell cycle profile, we used a CCI cutoff of 10 and identified 91 drugs with diverse cell cycle profiles (Fig. 2A and Supplementary Table 2). Among these 91 drugs, 30 were well-characterized cytotoxic anticancer drugs including doxorubicin (CCI = 77.03), paclitaxel (CCI = 57.84), and etoposide (CCI = 61.92) (Supplementary Table 2).

Next, we asked if the CCI correlated with a drugs cytotoxicity. First, we evaluated the cytotoxic effects of these 91 FDA-approved drugs in a cell viability assay. HeLa cancer cells were plated into 384-well plates and each drug was added at 50 μM final concentration. Seventy-two hours later, cell viability was determined using the CellTiterGlo assay. Of the 91 compounds, 46 reduced HeLa cell viability >3 standard deviations from the DMSO control while 38 compounds had less than 50% cell viability (Fig. 2B, Supplementary Fig. 1 and Supplementary Table 3). Importantly, the CCI showed a strong correlation with the extent of a compounds ability to reduce HeLa cell viability at this cutoff (Fig. 2B). We further evaluated the ability of the 46 hit compounds to reduce HeLa cell viability by performing a dose-dependent titration under the same conditions described above and determined that 36 compounds had an EC_50_ < 20 μM, which represented the most potent cytotoxic agents (Fig. 2C and Supplementary Table 4). The 36 identified compounds included 14 known anticancer drugs that reduced cancer cell viability and targeted tubulin or DNA and 22 compounds that were not originally indicated for the treatment of cancer (Fig. 2D)^25–37^. Among the 14 known anticancer drugs were tubulin targeting agents like microtubule stabilizers, paclitaxel (EC_50_ = 3.24 nM), parthenolide (EC_50_ = 17.33 nM) and destabilizers like colchicine (EC_50_ = 8.79 nM), parbendazole (EC_50_ = 0.53 μM), fenbendazole (EC_50_ = 2.27 μM) and mebendazole (EC_50_ = 3.39 μM). On the other hand, anticancer DNA damaging agents included compounds targeting the topoisomerase machinery such as mitoxantrone (EC_50_ < 0.01 μM), camptothecin (EC_50_ = 0.13 μM), etoposide (EC_50_ = 0.92 μM), and podophyllotoxin (EC_50_ = 0.38 μM), or direct DNA binders like doxorubicin (EC_50_ = 0.52 μM), daunorubicin (EC_50_ = 1.37 μM), chlorambucil (EC_50_ = 1.37 μM) and cyclohexamide (EC_50_ = 0.33 μM). Additionally, 22 of the identified compounds were not originally indicated for the treatment of cancer (Fig. 2D). Among these, 16 compounds had been previously demonstrated to have activity against multiple cancer cell lines including proscillaridin A (EC_50_ = 5 nM), diethylstilbestrol (EC_50_ = 0.15 µM), monensin (EC_50_ = 0.38 µM), cardiac glycosides such as digoxigenin, digoxin and lanatoside C (EC_50_ = 0.26 µM, 0.73 µM, 1.84 µM), norgestrel (EC_50_ = 1.45 µM), 17-beta estradiol (EC_50_ = 2.62 µM), niclosamide (EC_50_ = 2.81 µM), statins such as fluvastatin and simvastatin (EC_50_ = 3.04 µM, 10.44 µM), eburnamonine (EC_50_ = 8.74 µM), alexidine (EC_50_ = 8.81 µM), methylbenzethonium (EC_50_ = 9.98 µM), ciclopirox (EC_50_ = 11.14 µM), and luteolin (EC_50_ = 16.1 µM)^30, 32–36, 38–43^. Importantly, 6 FDA-approved drugs with novel cytotoxic effects were discovered including methiazole (EC_50_ = 1.37 µM), medrysone (EC_50_ = 9.13 µM), nicergoline (EC_50_ = 13.34 µM), tribenoside (EC_50_ = 13.74 µM), primaquine (EC_50_ = 16.16 µM), and GBR 12909 (EC_50_ = 17.91 µM) (Supplementary Fig. 2A). These 6 drugs also significantly decreased the cell viability of HCT116 (colon cancer), U2OS (bone osteosarcoma) and A549 (lung carcinoma) cancer cell lines, indicating that their cytotoxic activities were not limited to cervical cancer cells (Supplementary Fig. 2B). Interestingly, medrysone is a corticosteroid commonly used in optometry to treat eye inflammation^44^. On the other hand, nicergoline and GBR 12909 (Vanoxerine) are used for the treatment of senile dementia and cocaine dependency respectively, while primaquine is effective against malaria^45–47^.

**Figure 3:**
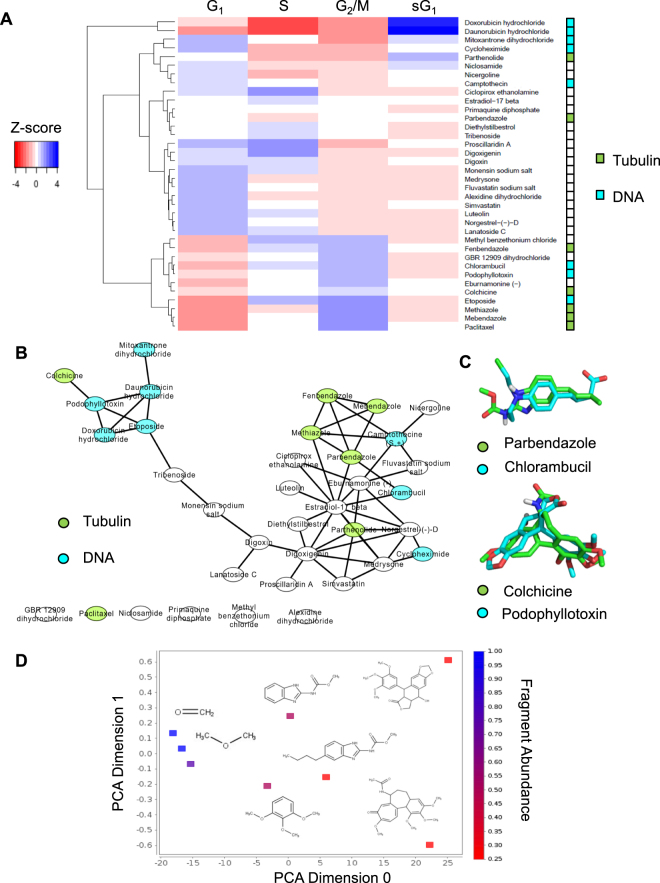
Computational cell cycle profiling and 3D chemical clustering for predicting drug activity. (**A**) The cell cycle profiles of the 36 most potent FDA-approved drugs that inhibited cancer cell viability were analyzed using hierarchical clustering and heatmap analyses. The four cell cycle phases (G_1_, S, G_2_/M and subG_1_) are indicated at the top of the hierarchical clustering heatmap. The relative percent arrest in each cell cycle phase was correlated with color intensity from red (low % arrest) to blue (high % arrest). Note that compounds with established tubulin targeting and DNA targeting mechanisms of action are indicated on the right side of the heatmap. The heatmap shows that compounds with similar mechanisms of action were clustered together based on unique cell cycle profile signatures. (**B**) CSNAP3D was used to perform computational network clustering of the 36 selected FDA-approved drugs that inhibited cancer cell viability based on 3D chemical similarity. The 3D coordinates of the 36 drugs were retrieved from PubChem. The molecular shape similarity between compounds was evaluated using the ShapeAlign algorithm to determine 3D chemical similarity scores. To visualize the pair-wise similarity relationship between drugs (nodes on the network), the computed adjacency matrix was mapped to the network structure using Cytoscape. As in (**A**), the compounds with established tubulin targeting and DNA targeting mechanisms of action are color coded as indicated. Note that many cell cycle profile similarity associations in (**A**), also showed ligand similarity associations. (**C**) Representative examples of ligand similarity pairs generated in (**B**). The 3D chemical structure alignments of the tubulin targeting agents parbendazole and colchicine and the DNA targeting agents chlorambucil and podophyllotoxin generated in (**B**) were visualized using PyMOL. (**D**) Chemical fingerprint analysis of four compounds (colchicine, parbendazole, methiazole and podophyllotoxin) linked to both microtubule and DNA damaging agents using the KNIME Analytics platform. The most common chemical fragments of the four drugs were identified using the MOSS algorithm. The identified consensus fragments were subsequently evaluated based on 36 chemical descriptors using the Chemistry Development (CDK) toolkit and clustered using the principal component analysis (PCA). The enriched chemical fragments were visualized using a scatterplot based on the primary (x-axis) and secondary (y-axis) principal components. The relative fragment abundance was then correlated with color intensity from red (low abundance) to blue (high abundance) as indicated on the right side of the scatter plot. Note that PCA determined that methoxy and carbonyl functional groups (blue squares and chemical structures depicted on scatter plot) were enriched in compounds linked to both microtubule and DNA damaging agents.

Since 22 out of the 36 most potent cytotoxic FDA-approved drugs were not originally indicated for the treatment of cancer, we sought to determine if their cell cycle profiles were similar to known anticancer agents; as a means to learn about the potential biological pathways that they were affecting. To do this, we clustered the cell cycle profiles of the 36 drugs using hierarchical clustering and heatmap analyses (Fig. 3A). A Euclidean distance metric was used to compute the similarity between drug cell cycle fingerprints followed by complete agglomerative clustering. The clustered cell cycle fingerprint profiles of the 36 drugs were then normalized by a Z-score transformation across the four cell cycle phases, G_1_, S, G_2_/M and subG_1_. As expected, compounds with similar and well characterized mechanisms of action were clustered based on common cell cycle profile signatures. For example, the DNA damaging agents doxorubicin and daunorubicin induced a similar increase in the subG_1_ cell population and were clustered near two other DNA binding agents mitoxantrone and cyclohexamide (Fig. 3A)^48^. On the other hand, podophyllotoxin, which inhibits both DNA replication and tubulin polymerization, clustered with the DNA targeting agent chlorambucil and the tubulin destabilizing agent colchicine (Fig. 3A). Interestingly, the heatmap also revealed unexpected links between several tubulin binding agents and DNA binding agents. Compounds like fenbendazole and colchicine, previously known for their specific tubulin destabilizing effects, induced a similar cell cycle profile to the DNA binders chlorambucil and etoposide. Cell cycle profile clustering also showed that methiazole, an antiworm drug, had cell cycle profile similarities to fenbendazole and etoposide, indicating that it could potentially induce microtubule and DNA damage^49^.

To further explore the significance of cell cycle profile similarities between drugs that induced microtubule damage and DNA damage, we analyzed the structural similarity of the 36 FDA-approved drugs using our recently developed three-dimensional Chemical Similarity Network Analysis Pulldown (CSNAP3D) algorithm, which clustered compounds into a chemical network based on the similarity of compound three-dimensional conformations^50, 51^. The basic assumption of this approach is the chemical similarity principle, which states that chemically similar compounds will have similar bioactivities. Thus, drugs with similar cell cycle profiles would likely cluster into the same subnetwork. Indeed, 3D chemical similarity clustering analysis of the 36 compounds indicated that many cell cycle profile similarity pairs shared 3D chemical similarities. For example, the chemical similarity network clustered doxorubicin, daunorubicin and mitoxantrone into a DNA binding subnetwork (Fig. 3B). Likewise, podophyllotoxin was simultaneously linked to the tubulin destabilizing agent colchicine and the DNA binding agents, doxorubicin, daunorubicin and etoposide, highlighting its established dual mechanism of action (Fig. 3B). Interestingly, methiazole was linked to three other tubulin destabilizing agents, fenbendazole, mebendazole and parbendazole as well as the known DNA binder camptothecin (Fig. 3B). Additionally, the tubulin destabilizers parbendazole and colchicine shared high 3D chemical similarity to the DNA targeting agents chlorambucil and podophyllotoxin, respectively (Fig. 3B and C). Importantly, the shared chemical similarity between these compounds could not be detected using simple chemical comparisons. For example, using their FP2 fingerprints to compute their 2D chemical similarity, the similarity between these compounds was low; 0.29 between parbendazole and chlorambucil and 0.34 between colchicine and podophyllotoxin. To identify the most commonly shared chemical motifs of compounds with potential links to microtubule and DNA damage (colchicine, methiazole, parbendazole and podophyllotoxin), we performed a chemical fragment enrichment analysis by clustering consensus molecular fragments using principal component analysis (PCA) (See Methods)^52^. Consistent with the structural alignment, PCA analysis showed that carbonyl and methoxy functional groups were the top two enriched chemical fragments of the four drugs (Fig. 3D). Notably, the methoxy functional group was shown to be essential for stabilizing the podophyllotoxin-topoisomerase complex as well as for podophyllotoxin binding to the colchicine site of beta tubulin^50, 53^. The presence of these functional groups offers novel insight into the future design of compounds that could be used to induce both microtubule and DNA damage.

**Figure 4:**
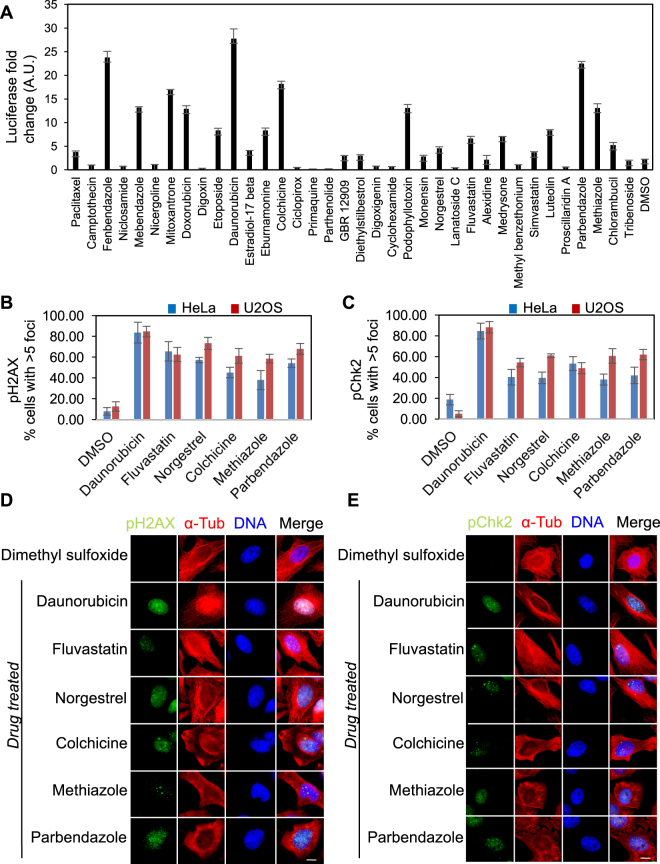
Novel associations of FDA-approved drugs with genotoxic stress and microtubule damage. (**A**) To determine if the 36 selected FDA-approved drugs were inducing DNA damage, each drug (at 50 μM) was tested for its ability to induce DNA damage in a genotoxic stress reporter cell line (HEK293 ATAD5-Luciferase) after 18 hours. Graph displays the average fold change in luciferase activity per cell (y-axis, expressed in arbitrary units) induced by the indicated drugs (x-axis) compared to the DMSO control. Error bars indicate standard deviations from 3 independent triplicate experiments. (**B**–**C**) HeLa or U2OS cells were treated with DMSO, Daunorubicin, Fluvastatin, Norgestrel, Colchicine, Methiazole, or Parbendazole at their respective EC_90s_ for 4 hours. Cells were then fixed and stained for DNA, α–tubulin, and the DNA damage markers phospho-Ser139-histone H2A.X (pH2AX) or phospho-Thr68-Chk2 (pChk2). Immunofluorescence microscopy was then used to quantify the percentage of non-mitotic cells with >5 pH2AX foci or pChk2 foci. Graphs display the percentage of non-mitotic cells with >5 pH2AX foci (**B**) or pChk2 foci (**C**) on the y-axis and the drug names on the x-axis. Bars in blue represent data for HeLa cells and bars in red represent data for U2OS cells. Error bars indicate standard deviations from 3 independent triplicate experiments. (**D**–**E**) Representative immunofluorescence microscopy images of HeLa cells that were treated with the indicated compounds at their respective EC_90s_ for 4 hours, fixed and stained for DNA, α–tubulin, and the DNA damage markers phospho-Ser139-histone H2A.X (pH2AX) (**B**) and phospho-Thr68-Chk2 (pChk2). (**C**). Scale bar = 5 μm.

Based on our cell cycle profile clustering and 3D chemical similarity clustering analyses of the 36 most potent FDA-approved drugs, we observed that many of these drugs shared cell cycle profile and 3D chemical similarities to DNA damaging agents. Therefore, we sought to determine whether they could induce DNA damage in a high-throughput genotoxicity assay. A HEK293T cell line that expressed luciferase-tagged ATAD5 in response to genotoxic stress was used as a reporter to test the ability of these compounds to induce DNA damage after an 18 hour drug treatment^54^. Interestingly, 4 tubulin destabilizing agents fenbendazole, mebendazole, parbendazole and colchicine revealed an unexpected potent DNA damage response (>5 fold) (Fig. 4A and Supplementary Table 5). Additionally, several non-anticancer drugs induced DNA damage to various levels relative to the DMSO control, including 17-beta estradiol (1.7 fold), eburnamonine (3.1 fold), norgestrel (2.5 fold), fluvastatin (3 fold), medrysone (2.8 fold), and luteolin (3 fold) (Fig. 4A and Supplementary Table 5). However, this high-throughput genotoxicity assay was conducted at 18 hours post drug treatment and previous studies have shown that DNA damage can arise in cells that have been arrested for prolonged lengths of time, including those that arrest in mitosis^55, 56^. Thus, it was possible that the DNA damage could have been caused by prolonged cell cycle arrests and not by a direct effect on the DNA. To ensure that the observed DNA damage was not an indirect effect of a prolonged drug-induced arrest, HeLa and U2OS cells were treated with 6 representative compounds (daunorubicin, fluvastatin, norgestrel, colchicine, methiazole and parbendazole) at their EC_90s_ for 4 hours and their ability to induce DNA and tubulin damage was assessed by immunostaining for tubulin and the DNA damage markers pH2AX and pCHK2 and by quantifying the percentage of non-mitotic cells with >5 pH2AX or pCHK2 foci^57, 58^. Consistent with our high throughput genotoxicity screen results, all six drugs induced DNA damage, as indicated by the increased immunostaining of pH2AX and pCHK2 and the increase in the percentage of HeLa and U2OS cells with >5 pH2AX or pCHK2 foci (Fig. 4B–E). Additionally, colchicine, methiazole and parbendazole also destabilized cytoplasmic microtubules, as can be seen by the lack of polymerized microtubules (Fig. 4B and C). Together these data indicated that tubulin destabilizing agents like colchicine, methiazole and parbendazole also induced DNA damage, which had been previously unreported.

## Discussion

The increased investment in cancer drug discovery to search for new chemical entities (NCE) has not translated into an increased development of new anticancer drugs. The majority of NCEs have failed in clinical trials due to their lack of efficacy and associated toxicities. Consequently, repurposing old drugs for new anticancer indications represents a viable alternative in the new paradigm of polypharmacology. The approved drugs guarantee sound pharmacological and safety profiles, and the discovery of new indications for a known agent can be quickly approved for clinical use. In the past, drug repurposing has often been the result of serendipitous discoveries. While drug repurposing for anticancer indications has recently been attempted, many of which have focused on a predefined mechanism or drug target, the diversity of repurposed drug classes has been limited.

In this study, we present a new cell cycle profiling approach for prioritizing FDA-approved drugs with repurposing potential. The approach assumes that a compound that inhibits cellular proliferation will perturb the cell cycle profile, leading to a different cell cycle distribution. Thus, the cell cycle profile induced by a compound can be used as an indicator of its antiproliferative effect. To quantify the cell cycle profile, we used DMSO treated cell cycle profiles as controls, and the Euclidean distances of chemical treated profiles were determined by the cell cycle index (CCI). Using this approach, we identified 46 compounds from the 884 FDA-approved drugs that showed an antiproliferative effect on HeLa cells. Among them, 36 compounds had an EC_50_ < 20 µM when evaluated in a cell viability assay including 6 compounds that showed novel antiproliferative effects on 4 different types of cancer cell lines. Although these compounds have EC_50s_ in the micro-molar range and are not ideal for therapeutic treatments in their current state, they provide potential opportunities for drug development.

Currently, major DNA-damaging drugs for the treatment of cancer include the DNA cross-linker cisplatin, the antimetabolite methotrexate, 5-fluorouracil (5-FU) and topoisomerase poisons, camptothecin and doxorubicin^59–61^. While these compounds are effective at treating a wide range of solid tumors and other malignancies, their uses are still limited by severe side-effect, dose limiting toxicities and the development of drug resistance^59^. Our cell cycle profiling analysis identified FDA-approved drugs that unexpectedly induced DNA damage, including several clinically relevant microtubule destabilizing agents like colchicine, methiazole and parbendazole, which have been widely employed in the clinical treatment of gout, familial Mediterranean fever and pericarditis, or used as anthelmintics for treating worm infections respectively^49, 62^. In particular, parbendazole and methiazole displayed similar cell cycle profiles to known DNA damaging agents and elicited a strong DNA damage response in genotoxicity assays. Our study suggests that a board class of microtubule destabilizing compounds could be investigated as inducers of microtubule and DNA damage to develop more effective therapies that target both microtubule and DNA pathways.

In conclusion, we have developed a new approach for prioritizing FDA-approved drugs as lead drugs for developing cancer therapeutics that relies on cell cycle profiling. Cell cycle profiling can potentially be integrated into computational flow cytometry workflows for large-scale phenotypic-based lead drug discovery. This approach could be expanded to include a board array of cancer cell lines to understand drug sensitivity and resistance. We envision that our drug cell cycle profiling approach could be universally applied to prioritize licensed drugs as lead drugs for the rapid development of cancer therapies that could potentially impact the quality of life and survival of cancer patients.

## Methods

### Cell Culture

HeLa, HCT116, U2OS, and A549 cell lines were purchased from the ATCC, which verified its identity by short-tandem repeat profiling, and cells were passaged for < 2 months following receipt. HeLa cells were maintained in F12:DMEM 50:50 medium, HCT116 and U2OS in McCoy’s 5 A medium, and A549 in DMEM medium (GIBCO) with 10% FBS, 2mM L-glutamine, and antibiotics (penicillin and streptomycin) in 5% CO_2_ at 37 °C.

### Cell Cytometry

HeLa cells were plated in 384-well plates (1500 cells/well) and treated with 10 µM drugs for 20 hours. Cells were fixed and stained with 5 μM Vybrant DyeCycle Green (Invitrogen) for 1 hour at room temperature and plates were scanned with an Acumen eX3 (TTP Labtech) fluorescence cytometer using a 488 nm laser and a cell cycle histogram profile was generated for each well. Data analysis was performed using the Collaborative Drug Discovery (CDD; www.collaborativedrug.com) software and outputs were exported to Excel. The quality of the screen was assessed by calculating the Z’ factor [Z’ factor = 1–3 x (*σ*_p_ + *σ*_n_)/(|*μ*_p_−*μ*_n_|)], which takes into account the dynamic range of the assay and variance of the data. The screen performed with an average plate Z’ factor of 0.51 ± 0.09, within the optimal performance range of 0.5–1.

### Computational Cell Cycle Profiling

The percentage of cells arrested in G_1_, S, G_2_/M and subG_1_ phases by each compound and DMSO was converted to cell cycle fingerprints < G_1_, S, G_2_/M, sG_1_ > and < G_10_, S_0_, G_2_/M_0_, sG_10_ > respectively where zero indicated the reference point. The relative distance between drug-induced and DMSO control was calculated by the expression < RG_1_, RS, RG_2_/M, RsG_1_ > = < G_1_–G_10_, S–S_0_, G_2_/M–G_2_/M_0_, sG_1_–sG_10_ > . The cell cycle index (CCI) defined by the Euclidean distance was obtained by

$$\mathrm{CCI}=\sqrt{R{{G}_{1}}^{2}+R{S}^{2}+R{G}_{2}{M}^{2}+Rs{{G}_{1}}^{2}}$$

For results of analyses, please see Supplementary Table S2.

### Cell Viability End-point Assays

20 µl of fresh DMEM:F12 medium was added to each well of a 384-well plate, 0.5 µl of drug stock was then plated into each well for a final drug concentration of 50 µM, and 30 µl of 5 × 10^4^ cells/ml HeLa cell suspension was added. Plates were incubated at room temperature for 30 minutes and then placed at 37 °C for 72 hours. After equilibrating at room temperature for approximately 30 minutes, 25 μl of CellTiterGlo® Reagent (Promega) was added to each well. Plates were incubated at room temperature for 10 minutes to stabilize the luminescent signal and luminescence was recorded using a Wallac plate reader (PerkinElmer). The average readout for the control DMSO-treated cells was used to calculate the average % cell viability of compound-treated cells. Similarly, HCT116, U2OS, and A549 cells were treated with the EC_90_ of each of the six selected drugs and cell viability was measured as described above.

### Compound Potency

An 8-point serial dilution (50000 µM, 12500 µM, 3125 µM, 781 µM, 260 µM, 65 µM, 16 µM, 4 µM) in DMSO was prepared for each test compound. HeLa cells were grown at 37 °C in 5% CO_2_ and 50:50 DMEM:F12 medium (GIBCO) supplemented with 10% fetal bovine serum and 1% antibiotics (penicillin and streptomycin). In each well of a 384-well plate, 20 µl of fresh medium was added, .5 µl of drug stock was then plated in each well for a final drug concentration of 50 µM to 0.04 µM, and 30 µl of 5 × 10^4^ cells/ml cell suspension was dispensed into each compound containing well in triplicate. Plates were incubated at room temperature for 30 minutes and placed at 37 °C for 72 hours. 25 μl of CellTiterGlo® reagent (Promega) was dispensed into each well. Plates were incubated at room temperature for 10 minutes to stabilize the luminescent signal. Luminescence was measured using a Wallac plate reader (PerkinElmer).

### Cell Cycle Profile Clustering Analysis

The cell cycle profile clustering analysis was conducted using the Heatmap.2 function in the R statistical package (version 3.4.1). Briefly, a Euclidean distance metric was used to compute the similarity between the 36 drug cell cycle fingerprints followed by the complete agglomerative clustering algorithm. The clustered cell cycle fingerprint profiles of the 36 drugs were then normalized by a Z-score transformation across the four cell cycle phases, G_1_, S, G_2_/M and subG_1_.

### Chemical Similarity Network Analysis

The chemical similarity network analysis was performed using the CSNAP3D (chemical similarity network analysis pull-down) 3D program^50, 51^. Briefly, the 3D coordinates of the 36 compounds with highest cell cycle index (CCI) were retrieved from the PubChem database. The molecular shape similarity between compounds was evaluated using the ShapeAlign algorithm to determine 3D chemical similarity scores and the generated 3D ligand alignments were analyzed using the PyMOL program^51^. To visualize the pair-wise similarity relationship between compounds, the computed adjacency matrix was mapped to the network structure using the Cytoscape program^63^.

### Chemical Fragment Enrichment Analysis

The chemical fragment enrichment analysis was performed using the KNIME Analytics platform^64^. Briefly, the most common chemical fragments of four drugs suspected of inducing microtubule and DNA damage (colchicine, parbendazole, methiazole and podophyllotoxin) were identified using the MOSS algorithm^65^. The identified consensus fragments were subsequently evaluated based on 36 chemical descriptors using the Chemistry Development (CDK) toolkit and clustered using the principal component analysis (PCA)^52, 66^. The enriched chemical fragments were visualized using a scatterplot based on the primary and secondary principal components.

### High-Throughput Genotoxic Assay

HEK293T ATAD5-luciferase cells were grown at 37 °C in 5% CO_2_ and 50:50 DMEM:F12 medium supplemented with 10% fetal bovine serum and 1% antibiotics. 1,500 cells were plated in each well of a 384 well plate, 20 ul of fresh medium was added to each well of a 384 well plate, followed by 0.5 μl of each drug at 5 mM or DMSO and 30 μl of 5 × 10^4^ cells/ml. The plates were incubated at 37 °C for 18 hours and equilibrated at room temperature for 30 minutes. To measure the ATAD5-luciferase activity, 50 ul of ONE-Glo® luciferase assay system reagent (Promega) was dispensed into each well and the luminescence signal was measured using a Wallac plate reader.

### Immunofluorescence Microcopy

Immunofluorescence microscopy was carried out as described^67^. HeLa or U2OS cells were treated with the indicated compounds for 4 hours, fixed, permeabilized, and co-stained with Hoechst 33342 (DNA stain) and the indicated antibodies. Images were captured with a Leica DMI6000 microscope (Leica DFC360 FX Camera, 63x/1.40-0.60 NA oil objective, Leica AF6000 software). Images were deconvolved with Leica Application Suite 3D Deconvolution software and exported as TIFF files.

### Antibodies

Immunofluorescence was carried out using the following antibodies: α-tubulin (Serotec: mca77g); phospho-histone H2A.X (Ser139) and phospho-Chk2 (Thr68) (Cell Signaling: 9718 S, 2661 S).

### Data Availability

All data generated or analyzed during this study are included in this published article and its Supplementary Information files or are available from the corresponding author on reasonable request.

## Electronic supplementary material

Supplementary Information
