# Supplementary material for: Computational Cell Cycle Profiling of Cancer Cells for Prioritizing FDA-Approved Drugs with Repurposing Potential
Source: Sci Rep. 2017 Sep 12;7:11261. doi: 10.1038/s41598-017-11508-2 (PMC5595967; doi:10.1038/s41598-017-11508-2)
Supplement: Supplementary file 1 — Supplementary Information [file 41598_2017_11508_MOESM1_ESM.pdf]

Supplementary Information

Computational Cell Cycle Profiling of Cancer Cells for Prioritizing FDA-Approved Drugs with Repurposing Potential

Yu-Chen Lo[1, 2], Silvia Senese[1], Bryan France[3,4], Ankur A. Gholkar[1], Robert Damoiseaux[3,4], Jorge Z. Torres[1, 5, 6*]

[1]Department of Chemistry and Biochemistry, University of California, Los Angeles, CA 90095.

[2]Program in Bioengineering, University of California, Los Angeles, CA 90095.

[3]Department of Molecular and Medical Pharmacology, Los Angeles, CA 90095.

[4]California NanoSystems Institute, University of California, Los Angeles, CA 90095.

[5]Jonsson Comprehensive Cancer Center, University of California, Los Angeles, CA 90095.

[6]Molecular Biology Institute, University of California, Los Angeles, CA 90095.

***Correspondence to:**

Jorge Z. Torres

UCLA Department of Chemistry and Biochemistry

Los Angeles, CA 90095

Phone: 310-206-2092

torres@chem.ucla.edu

Supplementary Figures

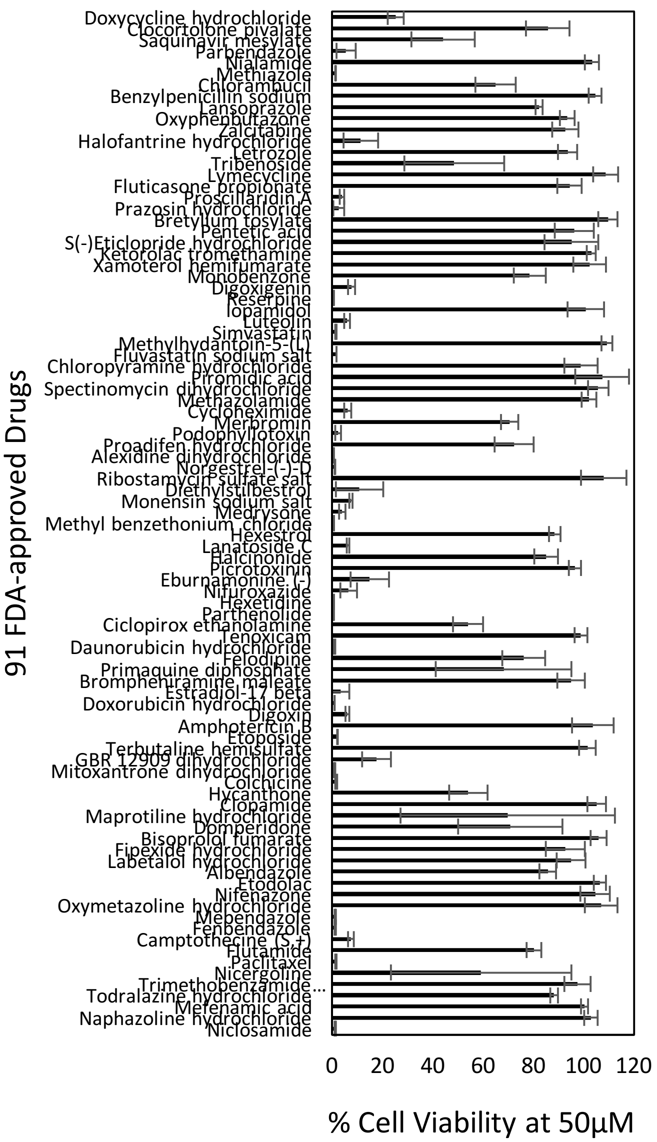

Supplementary Figure 1. Effect of FDA-approved drugs on HeLa cancer cell viability. 91 compounds with a cell cycle index (CCI) > 10 were evaluated for their antiproliferative effects using a cell viability assay. HeLa cells were treated with the indicated FDA-approved drugs (50µM) and cell viability was measured after 72 hours using the CellTiterGlo assay (See Methods). The cell viability measured by the luminescent signal was normalized against the DMSO control and converted to % cell viability.

A

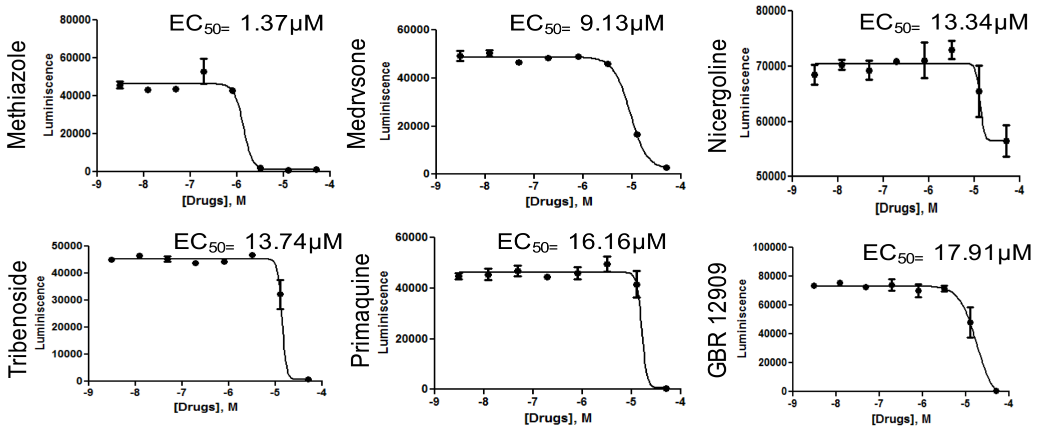

B

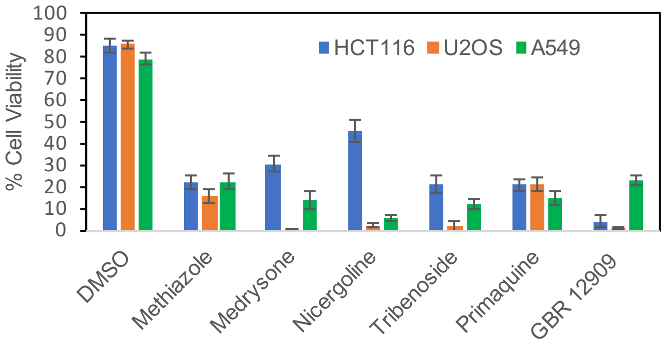

Supplementary Figure 2. Discovery of 6 novel FDA-approved drugs that reduce cancer cell viability. (A) HeLa cells were treated with increasing concentrations of the indicated FDA-approved drugs and cell viability was measured after 72 hours using the CellTiterGlo assay (See Methods). Graphs display cell viability on the y-axis (expressed in luminescence units) and increasing drug concentrations on the x-axis. The EC_{50s} for methiazole (1.37µM), medrysone (9.13µM), nicergoline (13.34µM), tribenoside (13.74µM), primaquine (16.16µM), and GBR 12909 (17.91µM) are indicated. (**B**) HCT116, U2OS and A549 cells were treated with the indicated compounds at their EC_{90s} and their cell viability was measured as indicated in **A**. Graph displays percent cell viability on the y-axis and drug treatments on the x-axis.

Supplementary Tables

Supplementary Table 1. Summary of high-throughput screening data.

Category	Parameter	Description
Assay	Type of assay	Cell-based
	Target	Cell cycle modulators
	Primary measurement	Cell cycle profile
	Key reagents	Vybrant DyeCycle Green Stain (Invitrogen)
	Assay protocol	See Supplementary Methods
	Additional comments	N/A
Library	Library size	884 compounds
	Library composition	FDA-approved drugs
	Source	UCLA Molecular Screening Shared Resource
	Additional comments	N/A
Screen	Format	384-well plates
	Concentration(s) tested	10µM, <1% DMSO
	Plate controls	Internal controls DMSO, Taxol
	Reagent/ compound dispensing system	Biomek FX (Beckman Coulter) and Multidrop 384 (Thermo LabSystems) liquid handlers
	Detection instrument and software	Acumen eX3 (TTP Labtech)
	Assay validation/QC	Z' score 0.51 ± 0.09
	Correction factors	N/A
	Normalization	To internal controls DMSO and Taxol
	Additional comments	N/A
Post-HTS analysis	Hit criteria	Cell cycle index (CCI) >10
	Hit rate	10.29%
	Additional assay(s)	Retesting top 91 compounds at 50µM for a decrease in >50% cell viability, Retesting top 46 compounds in a titration series to derive EC50s
	Confirmation of hit purity and structure	Compounds were repurchased from MolPort and compound structure and purity were verified analytically
	Additional comments	N/A

Supplementary Table 2. Cell cycle profiling of 884 FDA-approved drugs.

Supplementary Table 3. The effect on cell viability of 91 selected FDA-approved drugs with a cell cycle index >1 standard deviation from the mean CCI.

Supplementary Table 4. Cell viability EC_{50} of 46 selected FDA-approved drugs.

Supplementary Table 5. Evaluation of genotoxicity of 36 selected FDA-approved drugs.

Name	G1	S	G2M	subG1	RG1	RS	RG2/M	RsG1
(-) -Levobunolol hydrochloride	55.59	11.27	32.04	1.093	-1.01	1.76	2.28	-3.132
(-)-Eseroline fumarate salt	55.31	13.56	27.28	3.849	-1.29	4.05	-2.48	-0.376
(-)-Isoproterenol hydrochloride	52.93	10.02	33.33	3.712	-3.67	0.51	3.57	-0.513
(-)-MK 801 hydrogen maleate	58.85	9.82	30.92	0.406	2.25	0.31	1.16	-3.819
(+) -Levobunolol hydrochloride	56.98	10.54	31.6	0.798	0.38	1.03	1.84	-3.427
(+)-Isoproterenol (+)-bitartrate salt	58.41	10.59	29.6	1.402	1.81	1.08	-0.16	-2.823
(+,-)-Synephrine	58.96	11.48	26.3	3.259	2.36	1.97	-3.46	-0.966
(R) -Naproxen sodium salt	58.7	9.14	30.47	1.684	2.1	-0.37	0.71	-2.541
(R)-(+)-Atenolol	55.96	9.71	31.77	2.56	-0.64	0.2	2.01	-1.665
(R)-Propranolol hydrochloride	53.56	9.81	34.1	2.529	-3.04	0.3	4.34	-1.696
(S)-(-)-Atenolol	62.53	9.39	26.29	1.796	5.93	-0.12	-3.47	-2.429
(S)-(-)-Cycloserine	50.53	12.56	33.71	3.196	-6.07	3.05	3.95	-1.029
(S)-propranolol hydrochloride	59.52	9.15	27.26	4.075	2.92	-0.36	-2.5	-0.15
2-Aminobenzenesulfonamide	58.48	10.89	29.59	1.047	1.88	1.38	-0.17	-3.178
2-Chloropyrazine	52.17	11.3	32.65	3.874	-4.43	1.79	2.89	-0.351
lpha-Hydroxy-5-beta-androstan-17-c	59.65	8.9	30.24	1.207	3.05	-0.61	0.48	-3.018
6-Furfurylaminopurine	55.75	9.36	32.02	2.859	-0.85	-0.15	2.26	-1.366
Acebutolol hydrochloride	59.5	9.63	28.17	2.7	2.9	0.12	-1.59	-1.525
Aceclofenac	57.66	8.58	30.21	3.554	1.06	-0.93	0.45	-0.671
Acemetacin	56.07	11.78	26.44	5.704	-0.53	2.27	-3.32	1.479
Acenocoumarol	58.18	10.3	27.7	3.818	1.58	0.79	-2.06	-0.407
Acetaminophen	62.54	8.14	26.55	2.769	5.94	-1.37	-3.21	-1.456
Acetazolamide	58.88	7.88	29.53	3.699	2.28	-1.63	-0.23	-0.526
Acetohexamide	56.61	9.11	30.58	3.696	0.01	-0.4	0.82	-0.529
Acetopromazine maleate salt	53.34	10.26	32.74	3.664	-3.26	0.75	2.98	-0.561
Acetylsalicylsalicylic acid	56.62	11.01	28.27	4.092	0.02	1.5	-1.49	-0.133
Acyclovir	56.22	11.6	27.13	5.049	-0.38	2.09	-2.63	0.824
Adamantamine fumarate	53.66	10.42	32.36	3.565	-2.94	0.91	2.6	-0.66
nosine 5'-monophosphate monohyd	56.71	9.3	30.95	3.045	0.11	-0.21	1.19	-1.18
Adiphenine hydrochloride	51.19	13.72	32.27	2.819	-5.41	4.21	2.51	-1.406
Adrenosterone	60.02	9	29.63	1.35	3.42	-0.51	-0.13	-2.875
Albendazole	26.76	13.05	53.4	6.391	-29.84	3.54	23.64	2.166
Alclometasone dipropionate	58.2	12.33	28.1	1.36	1.6	2.82	-1.66	-2.865
Alcuronium chloride	57.22	12.11	27.76	2.906	0.62	2.6	-2	-1.319
Alexidine dihydrochloride	65.34	11.19	16.09	7.38	8.74	1.68	-13.67	3.155
Alfadolone acetate	52.97	12.53	30.69	3.806	-3.63	3.02	0.93	-0.419
Alfaxalone	58.44	11.44	26.67	3.457	1.84	1.93	-3.09	-0.768
Alfuzosin hydrochloride	58.89	9.89	28.62	2.602	2.29	0.38	-1.14	-1.623
Allantoin	52.19	12.48	31.96	3.292	-4.41	2.97	2.2	-0.933
Alprenolol hydrochloride	58.77	9.88	26.17	5.18	2.17	0.37	-3.59	0.955
Althiazide	55.62	9.43	30.71	4.152	-0.98	-0.08	0.95	-0.073
Alverine citrate salt	56.87	9.74	29.95	3.448	0.27	0.23	0.19	-0.777
Ambroxol hydrochloride	57.97	9.71	29.13	3.116	1.37	0.2	-0.63	-1.109
Amethopterin (R,S)	55.28	8.7	32.15	3.787	-1.32	-0.81	2.39	-0.438
Amidopyrine	57.56	11.1	28.38	2.957	0.96	1.59	-1.38	-1.268
Amikacin hydrate	51.97	10.82	33.61	3.525	-4.63	1.31	3.85	-0.7
Amiloride hydrochloride dihydrate	58.31	8.46	29.83	3.335	1.71	-1.05	0.07	-0.89
Aminocaproic acid	52.85	10.12	36.16	0.865	-3.75	0.61	6.4	-3.36

Aminohippuric acid	51.81	13.35	31.57	3.268	-4.79	3.84	1.81	-0.957
Aminophylline	61.4	9	28.18	1.421	4.8	-0.51	-1.58	-2.804
Aminopurine, 6-benzyl	53.7	9.73	33.31	3.262	-2.9	0.22	3.55	-0.963
Amiprilose hydrochloride	58.89	10.2	29.71	1.2	2.29	0.69	-0.05	-3.025
Amitryptiline hydrochloride	58.02	12.01	26.93	2.909	1.42	2.5	-2.83	-1.316
\modiaquin dihydrochloride dihydrate	58.16	9.25	28.67	3.863	1.56	-0.26	-1.09	-0.362
Amoxapine	58.8	10.66	25.5	5.043	2.2	1.15	-4.26	0.818
Amoxicillin	53.74	8.84	34.61	2.806	-2.86	-0.67	4.85	-1.419
Amphotericin B	50.81	9.24	26.1	13.856	-5.79	-0.27	-3.66	9.631
Ampicillin trihydrate	56.95	8.75	30.82	3.425	0.35	-0.76	1.06	-0.8
Amprolium hydrochloride	61.01	9.73	23.06	6.028	4.41	0.22	-6.7	1.803
Ampyrone	56.54	12.08	25.15	6.231	-0.06	2.57	-4.61	2.006
Amrinone	51.03	12.28	33.52	3.172	-5.57	2.77	3.76	-1.053
Amyleine hydrochloride	56.47	9.09	30.96	3.439	-0.13	-0.42	1.2	-0.786
Androsterone	54.48	9.56	32.32	3.638	-2.12	0.05	2.56	-0.587
Antazoline hydrochloride	56.72	9.35	29.22	4.707	0.12	-0.16	-0.54	0.482
Antimycin A	58.79	10.38	26.79	4.04	2.19	0.87	-2.97	-0.185
Antipyrine	58.12	12.04	25.26	4.422	1.52	2.53	-4.5	0.197
Antipyrine, 4-hydroxy	58.88	10.62	26.35	4.157	2.28	1.11	-3.41	-0.068
Apramycin	55	10.62	31.09	3.285	-1.6	1.11	1.33	-0.94
Artemisinin	52.51	9.95	35.12	2.422	-4.09	0.44	5.36	-1.803
Articaine hydrochloride	57.09	10.9	30.45	1.567	0.49	1.39	0.69	-2.658
Ascorbic acid	57.82	9.42	29.32	3.44	1.22	-0.09	-0.44	-0.785
Astemizole	61.25	8.53	27.02	3.192	4.65	-0.98	-2.74	-1.033
Atovaquone	53.06	8.74	35.22	2.973	-3.54	-0.77	5.46	-1.252
Atractyloside potassium salt	59.7	9.66	28.49	2.147	3.1	0.15	-1.27	-2.078
Atracurium besylate	56.82	7.44	31.98	3.752	0.22	-2.07	2.22	-0.473
Atropine sulfate monohydrate	56.38	8.95	31.29	3.369	-0.22	-0.56	1.53	-0.856
Azacyclonol	56.77	9.12	29.92	4.193	0.17	-0.39	0.16	-0.032
Azacytidine-5	55.94	12.97	29.8	1.287	-0.66	3.46	0.04	-2.938
Azaguanine-8	51.58	10.24	33.06	5.12	-5.02	0.73	3.3	0.895
Azaperone	59.66	10.76	24.45	5.124	3.06	1.25	-5.31	0.899
Azapropazone	57.26	11.13	29.29	2.318	0.66	1.62	-0.47	-1.907
Azathioprine	58.1	8.28	30.93	2.7	1.5	-1.23	1.17	-1.525
Azlocillin sodium salt	55.69	12.73	27.88	3.704	-0.91	3.22	-1.88	-0.521
Aztreonam	58.64	8.96	29.47	2.929	2.04	-0.55	-0.29	-1.296
Bacampicillin hydrochloride	55.51	10.29	31.1	3.102	-1.09	0.78	1.34	-1.123
Bacitracin	60.09	9.5	29.46	0.884	3.49	-0.01	-0.3	-3.341
Baclofen (R,S)	59.75	8.66	28.46	3.045	3.15	-0.85	-1.3	-1.18
Bambuterol hydrochloride	52.73	9.52	33.59	4.156	-3.87	0.01	3.83	-0.069
Beclomethasone dipropionate	54.5	13.4	31.12	0.978	-2.1	3.89	1.36	-3.247
Bemegride	63.61	8.12	26.32	1.868	7.01	-1.39	-3.44	-2.357
Bendroflumethiazide	60.63	10.08	27.46	1.825	4.03	0.57	-2.3	-2.4
Benfluorex hydrochloride	56.04	11.59	28.83	3.534	-0.56	2.08	-0.93	-0.691
Benfotiamine	62.58	9.26	26.96	1.12	5.98	-0.25	-2.8	-3.105
Benoxinate hydrochloride	53.74	8.9	34.18	3.182	-2.86	-0.61	4.42	-1.043
Benperidol	61.4	8.75	26.49	3.364	4.8	-0.76	-3.27	-0.861
Benserazide hydrochloride	57.77	10.09	31.02	1.121	1.17	0.58	1.26	-3.104
Benzathine benzylpenicillin	55.83	12.45	27.2	4.52	-0.77	2.94	-2.56	0.295

Benzbromarone	58.78	8.93	27.48	4.814	2.18	-0.58	-2.28	0.589
Benzethonium chloride	62.12	8.64	27.22	2.019	5.52	-0.87	-2.54	-2.206
Benzocaine	61.65	10.58	26.25	1.432	5.05	1.07	-3.51	-2.793
Benzonatate	61.09	9.88	25.21	3.818	4.49	0.37	-4.55	-0.407
Benzthiazide	61.96	10.44	26.01	1.594	5.36	0.93	-3.75	-2.631
Benzydamine hydrochloride	58.37	8.68	29.27	3.68	1.77	-0.83	-0.49	-0.545
Benzylpenicillin sodium	46.12	13.98	36.8	3.025	-10.48	4.47	7.04	-1.2
Bephenium hydroxynaphthoate	55.99	11.14	32.11	0.763	-0.61	1.63	2.35	-3.462
Bepridil hydrochloride	54.66	10.77	27.88	6.694	-1.94	1.26	-1.88	2.469
Beta-Escin	61.48	11.52	26.11	0.886	4.88	2.01	-3.65	-3.339
Betahistine mesylate	54.25	9.96	32.76	3.033	-2.35	0.45	3	-1.192
Betamethasone	62.97	6.44	27.15	3.451	6.37	-3.07	-2.61	-0.774
Betaxolol hydrochloride	56.16	12	27.76	4.082	-0.44	2.49	-2	-0.143
Betazole hydrochloride	58.79	9.02	29.79	2.352	2.19	-0.49	0.03	-1.873
Bethanechol chloride	49.74	12.63	35.65	1.884	-6.86	3.12	5.89	-2.341
Bezafibrate	52.8	11.41	32.07	3.73	-3.8	1.9	2.31	-0.495
Biotin	53.82	9.27	33.56	3.348	-2.78	-0.24	3.8	-0.877
Biperiden hydrochloride	58.64	9.19	28.13	4.041	2.04	-0.32	-1.63	-0.184
Bisacodyl	52.58	11.31	32.88	3.085	-4.02	1.8	3.12	-1.14
Bisoprolol fumarate	45.48	9.25	20.96	24.303	-11.12	-0.26	-8.8	20.078
Bretylium tosylate	64.98	8.35	25.12	1.555	8.38	-1.16	-4.64	-2.67
Brinzolamide	54.55	9.65	32.54	3.27	-2.05	0.14	2.78	-0.955
Bromocryptine mesylate	55.45	8.89	32.08	3.53	-1.15	-0.62	2.32	-0.695
Bromopride	60.28	9.5	26.4	3.818	3.68	-0.01	-3.36	-0.407
Bromperidol	57.67	8.71	30.67	2.955	1.07	-0.8	0.91	-1.27
Brompheniramine maleate	31.12	10.13	42.94	15.682	-25.48	0.62	13.18	11.457
Bucladesine sodium salt	64.81	8.44	25.47	1.281	8.21	-1.07	-4.29	-2.944
Budesonide	54.75	10.85	29.86	4.545	-1.85	1.34	0.1	0.32
Bufexamac	57.64	10.76	28.36	3.241	1.04	1.25	-1.4	-0.984
Buflomedil hydrochloride	54.27	9.4	32.82	3.504	-2.33	-0.11	3.06	-0.721
Bumetanide	54.96	12.74	28.31	3.983	-1.64	3.23	-1.45	-0.242
Bupivacaine hydrochloride	58.09	10.86	26.97	4.08	1.49	1.35	-2.79	-0.145
Bupropion hydrochloride	60.88	9.39	25.93	3.8	4.28	-0.12	-3.83	-0.425
Butacaine	62.14	8.78	28.09	0.994	5.54	-0.73	-1.67	-3.231
Butamben	58.71	11.51	25.23	4.549	2.11	2	-4.53	0.324
Butoconazole nitrate	56.69	9.41	31.19	2.712	0.09	-0.1	1.43	-1.513
Butylparaben	60.87	10.26	28.1	0.766	4.27	0.75	-1.66	-3.459
Camptothecine (S,+)	52.04	11.04	22.19	14.724	-4.56	1.53	-7.57	10.499
Canrenoic acid potassium salt	56.35	11.44	27.83	4.37	-0.25	1.93	-1.93	0.145
Captopril	56.23	9.18	31.79	2.739	-0.37	-0.33	2.03	-1.486
Carbachol	56.13	9.77	33.13	0.977	-0.47	0.26	3.37	-3.248
Carbamazepine	59.77	9.65	27.18	3.397	3.17	0.14	-2.58	-0.828
Carbarsone	57.86	9.26	29.48	3.398	1.26	-0.25	-0.28	-0.827
Carbenoxolone disodium salt	58.77	8.7	30.98	1.553	2.17	-0.81	1.22	-2.672
Carbetapentane citrate	58.05	11.12	27.22	3.61	1.45	1.61	-2.54	-0.615
Carbimazole	61.72	11.15	22.36	4.713	5.12	1.64	-7.4	0.488
Carbinoxamine maleate salt	60.99	9.23	28.1	1.678	4.39	-0.28	-1.66	-2.547
Carisoprodol	60.5	10.72	25.18	3.598	3.9	1.21	-4.58	-0.627
Carteolol hydrochloride	58.08	9.15	29.31	3.464	1.48	-0.36	-0.45	-0.761

Cefadroxil	52.79	11.03	33.08	3.097	-3.81	1.52	3.32	-1.128
Cefazolin sodium salt	60.57	9.34	28.9	1.108	3.97	-0.17	-0.86	-3.117
Cefepime hydrochloride	53.15	13.12	30.4	3.336	-3.45	3.61	0.64	-0.889
Cefixime	56.52	11.1	27.76	4.626	-0.08	1.59	-2	0.401
Cefmetazole sodium salt	61.57	10.79	25.85	1.786	4.97	1.28	-3.91	-2.439
Cefoperazone dihydrate	54.05	10.87	32.6	2.486	-2.55	1.36	2.84	-1.739
Ceforanide	57.01	10.41	26.98	5.604	0.41	0.9	-2.78	1.379
Cefotaxime sodium salt	59.47	9.37	27.51	3.647	2.87	-0.14	-2.25	-0.578
Cefotetan	56.9	8.14	31.28	3.671	0.3	-1.37	1.52	-0.554
Cefotiam hydrochloride	60.51	10.79	24.46	4.237	3.91	1.28	-5.3	0.012
Cefoxitin sodium salt	59.76	10.36	28.66	1.215	3.16	0.85	-1.1	-3.01
Cefsulodin sodium salt	59.95	9.91	28.35	1.787	3.35	0.4	-1.41	-2.438
Ceftazidime pentahydrate	52.92	11.23	32.56	3.219	-3.68	1.72	2.8	-1.006
Cefuroxime sodium salt	59.1	11.07	28.95	0.882	2.5	1.56	-0.81	-3.343
Cephalosporanic acid, 7-amino	55.56	10.14	30.78	3.453	-1.04	0.63	1.02	-0.772
Cephalothin sodium salt	54.69	8.75	35.16	1.406	-1.91	-0.76	5.4	-2.819
Cetirizine dihydrochloride	53.13	9.72	33.53	3.624	-3.47	0.21	3.77	-0.601
Chenodiol	56.92	9.07	30.56	3.389	0.32	-0.44	0.8	-0.836
Chicago sky blue 6B	54.05	9.09	33.88	2.975	-2.55	-0.42	4.12	-1.25
Chlorambucil	28.3	13.28	54.12	4.304	-28.3	3.77	24.36	0.079
Chloramphenicol	57.55	8.28	31.21	2.964	0.95	-1.23	1.45	-1.261
Chlorhexidine	57.32	8.07	29.52	5.086	0.72	-1.44	-0.24	0.861
Chlormezanone	60.09	10.09	25.34	4.472	3.49	0.58	-4.42	0.247
Chloropyramine hydrochloride	23.33	10.22	64.56	1.889	-33.27	0.71	34.8	-2.336
Chloroquine diphosphate	62.53	9.78	23.49	4.209	5.93	0.27	-6.27	-0.016
Chlorothiazide	59.5	10.23	26.37	3.897	2.9	0.72	-3.39	-0.328
Chlorotrianisene	56.5	10.09	31.4	2.018	-0.1	0.58	1.64	-2.207
Chlorpheniramine maleate	56.54	9.77	31.02	2.664	-0.06	0.26	1.26	-1.561
Chlorphensin carbamate	55.05	9.4	33.16	2.277	-1.55	-0.11	3.4	-1.948
Chlorpromazine hydrochloride	59.53	9.94	27.44	3.092	2.93	0.43	-2.32	-1.133
Chlorpropamide	54.39	9.93	32.86	2.812	-2.21	0.42	3.1	-1.413
Chlorprothixene hydrochloride	57.16	9.99	29.38	3.473	0.56	0.48	-0.38	-0.752
Chlortetracycline hydrochloride	59.95	9.88	25.45	4.716	3.35	0.37	-4.31	0.491
Chlorthalidone	59.85	8.29	27.65	4.144	3.25	-1.22	-2.11	-0.081
Chlorzoxazone	60.1	9.37	27.34	3.192	3.5	-0.14	-2.42	-1.033
Ciclopirox ethanolamine	61.04	18.36	17.87	2.734	4.44	8.85	-11.89	-1.491
Cimetidine	58.49	9.81	28.05	3.649	1.89	0.3	-1.71	-0.576
Cinnarizine	57.64	8.83	30.16	3.37	1.04	-0.68	0.4	-0.855
Cinoxacin	58.92	8.57	31.33	1.169	2.32	-0.94	1.57	-3.056
Ciprofibrate	52.39	13.33	31.94	2.347	-4.21	3.82	2.18	-1.878
Cisapride	60.41	10.93	25.1	3.563	3.81	1.42	-4.66	-0.662
Clebopride maleate	55.57	11.44	30.41	2.583	-1.03	1.93	0.65	-1.642
Clemastine fumarate	60.99	9.1	27.34	2.567	4.39	-0.41	-2.42	-1.658
Clemizole hydrochloride	53.66	8.41	34.02	3.902	-2.94	-1.1	4.26	-0.323
Clenbuterol hydrochloride	53.88	10.04	32.63	3.451	-2.72	0.53	2.87	-0.774
Clidinium bromide	61.81	9.84	27.46	0.894	5.21	0.33	-2.3	-3.331
Clindamycin hydrochloride	54.35	9.32	32.65	3.69	-2.25	-0.19	2.89	-0.535
Clioquinol	57.82	10.65	30.5	1.031	1.22	1.14	0.74	-3.194
Clocortolone pivalate	43.17	23.19	30.12	3.514	-13.43	13.7	0.36	-0.711

Clofazimine	62.48	11.34	22.35	3.834	5.88	1.83	-7.41	-0.391
Clofibric acid	60.47	8.94	29.41	1.176	3.87	-0.57	-0.35	-3.049
Clofilium tosylate	54.88	9.12	32.9	3.039	-1.72	-0.39	3.14	-1.186
Clomiphene citrate (Z,E)	53.89	9.82	32.49	3.794	-2.71	0.31	2.73	-0.431
Clomipramine hydrochloride	54.51	9.68	32.4	3.41	-2.09	0.17	2.64	-0.815
Clonidine hydrochloride	53.61	11.9	30.38	4.108	-2.99	2.39	0.62	-0.117
Clopamide	70.45	8.75	15.89	4.912	13.85	-0.76	-13.87	0.687
Cloperastine hydrochloride	62	9.99	23.49	4.522	5.4	0.48	-6.27	0.297
Clorgyline hydrochloride	56.63	9.04	29.58	4.751	0.03	-0.47	-0.18	0.526
Clorsulon	54.43	10.7	31.13	3.739	-2.17	1.19	1.37	-0.486
Clotrimazole	51.07	12.66	32.36	3.912	-5.53	3.15	2.6	-0.313
Cloxacillin sodium salt	57.49	9.45	28	5.069	0.89	-0.06	-1.76	0.844
Clozapine	62.33	10.54	23.95	3.178	5.73	1.03	-5.81	-1.047
Colchicine	26.42	10.06	54.24	8.962	-30.18	0.55	24.48	4.737
Colistin sulfate	59.79	9.26	27.05	3.899	3.19	-0.25	-2.71	-0.326
Corticosterone	58.78	9.69	27.67	3.859	2.18	0.18	-2.09	-0.366
Cortisone	61.06	7.64	28.37	2.814	4.46	-1.87	-1.39	-1.411
Cromolyn disodium salt	62.24	9.88	26.44	1.44	5.64	0.37	-3.32	-2.785
Crotamiton	56.94	8.98	33.09	0.997	0.34	-0.53	3.33	-3.228
Cyanocobalamin	51.21	11.84	33.33	3.543	-5.39	2.33	3.57	-0.682
Cyclizine hydrochloride	54.91	8.92	32.78	3.386	-1.69	-0.59	3.02	-0.839
Cyclobenzaprine hydrochloride	58.26	9.86	27.89	3.984	1.66	0.35	-1.87	-0.241
Cycloheximide	77	6.83	11.83	4.336	20.4	-2.68	-17.93	0.111
Cyclopenthiazide	56.2	11.95	28.85	3.005	-0.4	2.44	-0.91	-1.22
Cyclopentolate hydrochloride	53.92	10.33	32.67	3.077	-2.68	0.82	2.91	-1.148
Cyclosporin A	56.1	8.97	30.59	4.338	-0.5	-0.54	0.83	0.113
Cyproheptadine hydrochloride	58.12	9.01	29.37	3.504	1.52	-0.5	-0.39	-0.721
Cyproterone acetate	61.24	6.72	29.42	2.625	4.64	-2.79	-0.34	-1.6
Dacarbazine	57.84	12.49	26.04	3.632	1.24	2.98	-3.72	-0.593
Danazol	53.53	9.76	28.6	8.033	-3.07	0.25	-1.16	3.808
Dantrolene sodium salt	54.7	10.2	32.51	2.596	-1.9	0.69	2.75	-1.629
Dapsone	51.54	9.53	36.07	2.863	-5.06	0.02	6.31	-1.362
Daunorubicin hydrochloride	14.4	1.01	0	84.584	-42.2	-8.5	-29.76	80.359
D-cycloserine	54.71	13.06	29.45	2.771	-1.89	3.55	-0.31	-1.454
Debrisoquin sulfate	57.54	9.49	29.42	3.551	0.94	-0.02	-0.34	-0.674
Decamethonium bromide	57.23	9.8	30.65	2.326	0.63	0.29	0.89	-1.899
Deferoxamine mesylate	61.63	9.85	27.52	1.008	5.03	0.34	-2.24	-3.217
Dehydrocholic acid	51.66	10.62	33.68	3.97	-4.94	1.11	3.92	-0.255
Dehydroisoandosterone 3-acetate	62.68	6.42	29.66	1.252	6.08	-3.09	-0.1	-2.973
Demecarium bromide	57.56	10.13	29.82	2.412	0.96	0.62	0.06	-1.813
Demeclocycline hydrochloride	53.92	9.39	32.11	4.587	-2.68	-0.12	2.35	0.362
Denatonium benzoate	52.75	14.96	28.51	3.78	-3.85	5.45	-1.25	-0.445
Deoxycorticosterone	58.94	11.79	27.76	1.521	2.34	2.28	-2	-2.704
Deptropine citrate	50.57	11.98	34.97	2.487	-6.03	2.47	5.21	-1.738
Dequalinium dichloride	56.84	10.44	27.51	5.22	0.24	0.93	-2.25	0.995
Desipramine hydrochloride	55	7.83	33.5	3.667	-1.6	-1.68	3.74	-0.558
Dexamethasone acetate	59.59	6.34	28.94	5.132	2.99	-3.17	-0.82	0.907
omethorphan hydrobromide monohy	53.48	9.29	34.44	2.786	-3.12	-0.22	4.68	-1.439
Dibucaine	57.6	9.9	29.03	3.473	1	0.39	-0.73	-0.752

Dichlorphenamide	61.22	10.02	27.99	0.771	4.62	0.51	-1.77	-3.454
Diclofenac sodium	53.92	10.81	31.13	4.136	-2.68	1.3	1.37	-0.089
Dicumarol	52.52	12.52	30.74	4.148	-4.08	3.01	0.98	-0.077
Dicyclomine hydrochloride	58.1	10.06	28.16	3.563	1.5	0.55	-1.6	-0.662
Dienestrol	53.68	10.11	32.53	3.607	-2.92	0.6	2.77	-0.618
Diethylcarbamazine citrate	55.31	9.06	32.21	3.419	-1.29	-0.45	2.45	-0.806
Diethylstilbestrol	45	14.13	38.57	2.302	-11.6	4.62	8.81	-1.923
Diflorasone Diacetate	58.84	9.82	28.48	2.857	2.24	0.31	-1.28	-1.368
Diflunisal	55.96	8.88	32.55	2.606	-0.64	-0.63	2.79	-1.619
Digitoxigenin	57.9	13.47	21.76	6.865	1.3	3.96	-8	2.64
Digoxigenin	58.61	16.94	22.13	2.322	2.01	7.43	-7.63	-1.903
Digoxin	59.48	13.6	20.6	6.319	2.88	4.09	-9.16	2.094
Dihydroergotamine tartrate	54.59	9.68	30.26	5.478	-2.01	0.17	0.5	1.253
Dihydrostreptomycin sulfate	56.09	9.95	31.33	2.573	-0.51	0.44	1.57	-1.652
Dilazep dihydrochloride	56.68	8.25	31.69	3.383	0.08	-1.26	1.93	-0.842
Diloxanide furoate	60.04	8.44	29.92	1.604	3.44	-1.07	0.16	-2.621
Dimaprit dihydrochloride	62.28	10.91	23.92	2.89	5.68	1.4	-5.84	-1.335
Dimenhydrinate	51.94	12.73	31.94	3.381	-4.66	3.22	2.18	-0.844
Dimethadione	58.08	10.98	29.85	1.005	1.48	1.47	0.09	-3.22
Dimethisoquin hydrochloride	57.31	9.3	29.24	4.152	0.71	-0.21	-0.52	-0.073
Dinoprost trometamol	49.91	12.5	34.35	3.241	-6.69	2.99	4.59	-0.984
Dioxybenzone	61.07	9.7	27.76	1.48	4.47	0.19	-2	-2.745
Diperodon hydrochloride	58.58	9.98	28.06	3.326	1.98	0.47	-1.7	-0.899
Diphemanil methylsulfate	55.34	9.46	32.14	3.074	-1.26	-0.05	2.38	-1.151
Diphenhydramine hydrochloride	54.13	9.91	32.66	3.303	-2.47	0.4	2.9	-0.922
Diphenidol hydrochloride	60.04	9.34	27.8	2.821	3.44	-0.17	-1.96	-1.404
Diphenylpyraline hydrochloride	53.98	13.08	28.49	4.456	-2.62	3.57	-1.27	0.231
Dipivefrin hydrochloride	54.47	8.78	34.06	2.693	-2.13	-0.73	4.3	-1.532
Diprophylline	54.43	9.25	33.71	2.611	-2.17	-0.26	3.95	-1.614
Dipyrone	52.69	13.24	31.38	2.69	-3.91	3.73	1.62	-1.535
Dirithromycin	51.43	11.02	34.68	2.867	-5.17	1.51	4.92	-1.358
Disopyramide	55.77	7.19	32.37	4.668	-0.83	-2.32	2.61	0.443
Disulfiram	58.25	10.2	28.83	2.721	1.65	0.69	-0.93	-1.504
Dizocilpine maleate	59.63	9.3	27.95	3.062	3.03	-0.21	-1.81	-1.163
DMSO	56.6	9.51	29.76	4.225	0	0	0	0
DO 897/99	56.74	9.14	31.85	2.263	0.14	-0.37	2.09	-1.962
Dobutamine hydrochloride	54.78	9.8	31.89	3.534	-1.82	0.29	2.13	-0.691
Domperidone maleate	43.26	15.21	37.89	3.552	-13.34	5.7	8.13	-0.673
Dorzolamide hydrochloride	52.89	12.92	32.06	2.126	-3.71	3.41	2.3	-2.099
Dosulepin hydrochloride	57.2	9.74	29.12	3.942	0.6	0.23	-0.64	-0.283
Doxazosin mesylate	61.99	8.89	27.98	1.142	5.39	-0.62	-1.78	-3.083
Doxepin hydrochloride	52.25	9.96	34.27	3.514	-4.35	0.45	4.51	-0.711
Doxorubicin hydrochloride	29.74	0.23	0.47	69.555	-26.86	-9.28	-29.29	65.33
Doxycycline hydrochloride	64.57	11.86	22.4	1.171	7.97	2.35	-7.36	-3.054
Doxylamine succinate	56.31	10.98	28.97	3.738	-0.29	1.47	-0.79	-0.487
Drofenine hydrochloride	60.68	9.46	25.5	4.361	4.08	-0.05	-4.26	0.136
Droperidol	59.43	11.05	25.32	4.199	2.83	1.54	-4.44	-0.026
Dropropizine (R,S)	57.69	11.7	25.94	4.679	1.09	2.19	-3.82	0.454
Dyclonine hydrochloride	58.61	9.77	26.98	4.644	2.01	0.26	-2.78	0.419

Dydrogesterone	59.53	8.97	30.29	1.206	2.93	-0.54	0.53	-3.019
Ebselen	50.37	13.41	32.67	3.556	-6.23	3.9	2.91	-0.669
Eburnamonine (-)	26.9	10.73	53.94	8.152	-29.7	1.22	24.18	3.927
Econazole nitrate	57.28	8.4	31.12	3.136	0.68	-1.11	1.36	-1.089
Edrophonium chloride	55.98	10.44	30.45	3.125	-0.62	0.93	0.69	-1.1
Enalapril maleate	52.95	8.72	35.7	2.635	-3.65	-0.79	5.94	-1.59
Enilconazole	60.28	10.46	25.93	3.241	3.68	0.95	-3.83	-0.984
Enoxacin	50.98	9.1	37.3	2.623	-5.62	-0.41	7.54	-1.602
Epiandrosterone	54.63	8.68	33.17	3.522	-1.97	-0.83	3.41	-0.703
Epirizole	58.95	9.17	27.43	4.45	2.35	-0.34	-2.33	0.225
Epitiostanol	57.38	9.62	27.79	5.216	0.78	0.11	-1.97	0.991
Equilin	60.42	10.6	27.36	1.63	3.82	1.09	-2.4	-2.595
Erythromycin	57.1	10.02	30.01	2.864	0.5	0.51	0.25	-1.361
Estradiol-17 beta	44.51	13.96	32.17	9.352	-12.09	4.45	2.41	5.127
Estriol	54.01	11.2	31.52	3.261	-2.59	1.69	1.76	-0.964
Estrone	62.33	10.43	26.35	0.884	5.73	0.92	-3.41	-3.341
Estropipate	63.71	9.92	24.88	1.488	7.11	0.41	-4.88	-2.737
Etanidazole	62.38	9.23	27.92	0.462	5.78	-0.28	-1.84	-3.763
Ethacrynic acid	52.72	11.22	30.13	5.857	-3.88	1.71	0.37	1.632
Ethambutol dihydrochloride	59.93	10.42	26.3	3.358	3.33	0.91	-3.46	-0.867
Ethamivan	52.42	9.39	35.78	2.415	-4.18	-0.12	6.02	-1.81
Ethamsylate	56.42	9.83	30.42	3.328	-0.18	0.32	0.66	-0.897
Ethaverine hydrochloride	63.36	10.32	25.53	0.788	6.76	0.81	-4.23	-3.437
Ethionamide	56.68	10.38	28.3	4.637	0.08	0.87	-1.46	0.412
Ethisterone	56.18	9.68	29.48	4.655	-0.42	0.17	-0.28	0.43
Ethopropazine hydrochloride	58.53	10.97	29.54	0.961	1.93	1.46	-0.22	-3.264
Ethosuximide	56.58	11.82	27.36	4.238	-0.02	2.31	-2.4	0.013
Ethotoin	59.89	10.48	27.68	1.955	3.29	0.97	-2.08	-2.27
Ethoxyquin	59.84	6.67	31.41	2.088	3.24	-2.84	1.65	-2.137
Ethynodiol diacetate	56.83	10.2	31.79	1.18	0.23	0.69	2.03	-3.045
Ethynylestradiol 3-methyl ether	61.78	8.8	28.47	0.952	5.18	-0.71	-1.29	-3.273
Etidronic acid, disodium salt	65.09	7.88	25.87	1.161	8.49	-1.63	-3.89	-3.064
Etifenin	58.66	9.44	28.62	3.271	2.06	-0.07	-1.14	-0.954
Etilefrine hydrochloride	51.25	13.86	33.39	1.502	-5.35	4.35	3.63	-2.723
Etodolac	39.7	18.23	39.99	2.017	-16.9	8.72	10.23	-2.208
Etofenamate	52.25	10.85	33.49	3.333	-4.35	1.34	3.73	-0.892
Etofylline	53.11	12.08	30.31	4.501	-3.49	2.57	0.55	0.276
Etomidate	54.67	11.35	31.08	2.899	-1.93	1.84	1.32	-1.326
Etoposide	11.12	15.33	71.32	2.225	-45.48	5.82	41.56	-2
Eucatropine hydrochloride	56.78	10.77	31.58	0.877	0.18	1.26	1.82	-3.348
Famotidine	58.38	10.03	28.87	2.721	1.78	0.52	-0.89	-1.504
Famprofazone	62.06	8.77	27.86	1.3	5.46	-0.74	-1.9	-2.925
Felbinac	59	11.5	25.66	3.834	2.4	1.99	-4.1	-0.391
Felodipine	47.53	10.93	38.06	3.482	-9.07	1.42	8.3	-0.743
Fenbendazole	20.43	13.71	58.38	6.599	-36.17	4.2	28.62	2.374
Fenbufen	58.72	11.89	25.14	4.251	2.12	2.38	-4.62	0.026
Fendiline hydrochloride	54.4	12.41	29.11	4.09	-2.2	2.9	-0.65	-0.135
Fenofibrate	52.62	13.46	30.71	3.202	-3.98	3.95	0.95	-1.023
Fenoprofen calcium salt dihydrate	56.27	9.72	33.28	0.725	-0.33	0.21	3.52	-3.5

Fenoterol hydrobromide	56.73	11.09	28.33	3.854	0.13	1.58	-1.43	-0.371
Fenspiride hydrochloride	54.22	10.07	33.74	1.968	-2.38	0.56	3.98	-2.257
Finasteride	58.13	10.89	25.54	5.445	1.53	1.38	-4.22	1.22
Fipexide hydrochloride	59.91	11.88	14.91	13.299	3.31	2.37	-14.85	9.074
Flavoxate hydrochloride	61.37	8.59	26.88	3.152	4.77	-0.92	-2.88	-1.073
Florfenicol	51.44	8.23	36.73	3.601	-5.16	-1.28	6.97	-0.624
Flucloxacillin sodium	58.1	10.24	29.12	2.542	1.5	0.73	-0.64	-1.683
Flucytosine	59.06	9.86	29.41	1.669	2.46	0.35	-0.35	-2.556
Fludrocortisone acetate	58.47	8.84	28.79	3.903	1.87	-0.67	-0.97	-0.322
Flumequine	52.85	11.94	30.66	4.545	-3.75	2.43	0.9	0.32
Flumethasone	55.94	13.98	28.39	1.682	-0.66	4.47	-1.37	-2.543
Flunisolide	58.78	8.73	27.97	4.514	2.18	-0.78	-1.79	0.289
Flunixin meglumine	63.32	9.42	26.17	1.09	6.72	-0.09	-3.59	-3.135
Fluocinonide	53.97	13.28	30.13	2.533	-2.63	3.77	0.37	-1.692
Fluorometholone	61.2	8.15	29.2	1.455	4.6	-1.36	-0.56	-2.77
Fluoxetine hydrochloride	58.88	9.96	25.5	5.659	2.28	0.45	-4.26	1.434
Fluphenazine dihydrochloride	51.59	10.76	34.94	2.646	-5.01	1.25	5.18	-1.579
Flurandrenolide	62.02	8.41	27.4	2.176	5.42	-1.1	-2.36	-2.049
Flurbiprofen	56.66	11.87	30.48	0.995	0.06	2.36	0.72	-3.23
Fluspirilen	64.63	7.66	25.77	1.935	8.03	-1.85	-3.99	-2.29
Flutamide	29.03	27.76	39.49	3.715	-27.57	18.3	9.73	-0.51
Fluticasone propionate	41.38	23.58	31.33	3.712	-15.22	14.1	1.57	-0.513
Fluvastatin sodium salt	67.15	11.49	20.88	0.479	10.55	1.98	-8.88	-3.746
Fluvoxamine maleate	54.3	11.19	32.42	2.094	-2.3	1.68	2.66	-2.131
Folic acid	49.46	11.4	36.27	2.873	-7.14	1.89	6.51	-1.352
Folinic acid calcium salt	52.21	11.93	32.03	3.76	-4.39	2.42	2.27	-0.465
Fosfosal	60.58	9.22	26.6	3.606	3.98	-0.29	-3.16	-0.619
Furaltadone hydrochloride	61.87	8.9	27.82	1.41	5.27	-0.61	-1.94	-2.815
Furazolidone	58.39	9.39	31.12	1.109	1.79	-0.12	1.36	-3.116
Furosemide	56.91	10.37	28.94	3.698	0.31	0.86	-0.82	-0.527
Fursultiamine Hydrochloride	53.85	12.79	30.41	2.951	-2.75	3.28	0.65	-1.274
Fusidic acid sodium salt	56.35	8.68	31.84	3.131	-0.25	-0.83	2.08	-1.094
Gabapentin	55.04	10.58	33.53	0.847	-1.56	1.07	3.77	-3.378
Gabexate mesilate	53.22	14.01	29.94	2.833	-3.38	4.5	0.18	-1.392
Galanthamine hydrobromide	58.62	11.71	24.57	5.101	2.02	2.2	-5.19	0.876
Gallamine triethiodide	53.26	11.18	31.46	4.097	-3.34	1.67	1.7	-0.128
Ganciclovir	58.57	10.78	29.52	1.126	1.97	1.27	-0.24	-3.099
GBR 12909 dihydrochloride	30.37	10.85	54.45	4.338	-26.23	1.34	24.69	0.113
Gemfibrozil	55.69	10.86	29.74	3.717	-0.91	1.35	-0.02	-0.508
Gentamicine sulfate	55.62	10.7	30.13	3.548	-0.98	1.19	0.37	-0.677
Glafenine hydrochloride	55.02	9.47	32.04	3.406	-1.58	-0.04	2.28	-0.819
Gliclazide	58.77	12.57	24.62	4.037	2.17	3.06	-5.14	-0.188
Glimepiride	60.9	9.11	28.8	1.196	4.3	-0.4	-0.96	-3.029
Gliquidone	52.78	13.03	31.77	2.416	-3.82	3.52	2.01	-1.809
Glutethimide, para-amino	61.58	9.35	25.16	3.797	4.98	-0.16	-4.6	-0.428
Glycopyrrolate	62.09	10.21	25.94	1.765	5.49	0.7	-3.82	-2.46
Griseofulvin	55.58	9.09	31.12	4.216	-1.02	-0.42	1.36	-0.009
Guaifenesin	57.67	9.43	31.39	1.438	1.07	-0.08	1.63	-2.787
Guanabenz acetate	58.74	9.12	25.64	6.427	2.14	-0.39	-4.12	2.202

Guanadrel sulfate	58.65	8.96	31.23	1.154	2.05	-0.55	1.47	-3.071
Guanethidine sulfate	56.58	9.24	31.41	2.714	-0.02	-0.27	1.65	-1.511
Guanfacine hydrochloride	55.53	10.33	31.87	2.271	-1.07	0.82	2.11	-1.954
Halcinonide	48.96	17.15	32.51	1.288	-7.64	7.64	2.75	-2.937
Halofantrine hydrochloride	48.45	13.59	36.18	1.708	-8.15	4.08	6.42	-2.517
Haloperidol	56.06	8.21	31.66	4.02	-0.54	-1.3	1.9	-0.205
Hemicholinium bromide	52.88	10.63	32.29	4.119	-3.72	1.12	2.53	-0.106
Heptaminol hydrochloride	60.29	10.01	25.17	4.52	3.69	0.5	-4.59	0.295
Hesperidin	58.98	8.87	28.78	3.381	2.38	-0.64	-0.98	-0.844
Hexamethonium dibromide dihydrate	54.48	8.78	32.65	4.082	-2.12	-0.73	2.89	-0.143
Hexestrol	48.1	12.97	36.12	2.807	-8.5	3.46	6.36	-1.418
Hexetidine	47.98	13.06	35.46	3.503	-8.62	3.55	5.7	-0.722
Hexylcaine hydrochloride	60.2	11.04	27.4	1.37	3.6	1.53	-2.36	-2.855
Homatropine hydrobromide (R,S)	57.4	10.8	29.18	2.625	0.8	1.29	-0.58	-1.6
Homochlorcyclizine dihydrochloride	56.35	10.42	30.39	2.732	-0.25	0.91	0.63	-1.493
Homosalate	56.84	12.44	28.3	2.421	0.24	2.93	-1.46	-1.804
Hycanthone	34.31	12.38	49.32	3.996	-22.29	2.87	19.56	-0.229
Hydralazine hydrochloride	51.62	10.81	31.98	5.588	-4.98	1.3	2.22	1.363
Hydrochlorothiazide	56.58	8.95	30.76	3.715	-0.02	-0.56	1	-0.51
Hydrocortisone base	50.5	12.77	28.89	7.839	-6.1	3.26	-0.87	3.614
Hydroflumethiazide	52.46	12.46	31.45	3.551	-4.14	2.95	1.69	-0.674
Hydroxytacrine maleate (R,S)	58.66	9.59	28.03	3.717	2.06	0.08	-1.73	-0.508
Hydroxyzine dihydrochloride	54.73	10.34	31.83	3.096	-1.87	0.83	2.07	-1.129
Hymecromone	59.09	9.71	29.79	1.328	2.49	0.2	0.03	-2.897
Hyoscyamine (L)	56.55	8.36	32.63	2.456	-0.05	-1.15	2.87	-1.769
Idazoxan hydrochloride	53.85	10.07	34.98	1.099	-2.75	0.56	5.22	-3.126
Idoxuridine	57.78	10.11	28.56	3.548	1.18	0.6	-1.2	-0.677
Ifenprodil tartrate	52.84	9.11	34.62	3.359	-3.76	-0.4	4.86	-0.866
Ifosfamide	58.53	10.54	29.87	1.061	1.93	1.03	0.11	-3.164
Imidurea	52.48	11.55	30.94	5.033	-4.12	2.04	1.18	0.808
Imipenem	54.53	9.14	33.42	2.909	-2.07	-0.37	3.66	-1.316
Imipramine hydrochloride	59.71	9.74	26.08	4.47	3.11	0.23	-3.68	0.245
Indapamide	59.81	8.72	28.21	3.197	3.21	-0.79	-1.55	-1.028
Indomethacin	60.45	10.42	25.35	3.782	3.85	0.91	-4.41	-0.443
Indoprofen	51.89	10.62	33.33	4.157	-4.71	1.11	3.57	-0.068
Iobenguane sulfate	57.21	10.22	29.84	2.721	0.61	0.71	0.08	-1.504
Iocetamic acid	60.11	10.69	28.05	1.158	3.51	1.18	-1.71	-3.067
Iodipamide	63.08	9.89	26.17	0.857	6.48	0.38	-3.59	-3.368
Iodixanol	62.68	9.35	26.74	1.226	6.08	-0.16	-3.02	-2.999
Iohexol	58.4	7.48	31.49	2.639	1.8	-2.03	1.73	-1.586
Iopamidol	50	13.29	35.92	0.787	-6.6	3.78	6.16	-3.438
Iopanoic acid	63.68	8.9	24.31	3.113	7.08	-0.61	-5.45	-1.112
Iopromide	60.57	8.54	29.61	1.277	3.97	-0.97	-0.15	-2.948
Ioversol	59.42	9.46	29.82	1.297	2.82	-0.05	0.06	-2.928
Ioxaglic acid	59.84	10.32	26.93	2.913	3.24	0.81	-2.83	-1.312
Iproniazide phosphate	57.67	8.6	30.9	2.83	1.07	-0.91	1.14	-1.395
Isocarboxazid	52.73	9.2	34.9	3.164	-3.87	-0.31	5.14	-1.061
Isoconazole	55.79	8.57	31.9	3.735	-0.81	-0.94	2.14	-0.49
Isoetharine mesylate salt	57.75	9.03	31.71	1.518	1.15	-0.48	1.95	-2.707

Isoflupredone acetate	63.62	6.57	26.34	3.472	7.02	-2.94	-3.42	-0.753
Isometheptene mucate	54.87	13.44	28.34	3.359	-1.73	3.93	-1.42	-0.866
Isoniazid	52.16	11.34	32.58	3.918	-4.44	1.83	2.82	-0.307
Isopropamide iodide	61.89	8.75	27.41	1.945	5.29	-0.76	-2.35	-2.28
Isopyrin hydrochloride	62.56	8.64	27.2	1.6	5.96	-0.87	-2.56	-2.625
-dimethoxy-1-methyl-1,2,3,4-tetrahyc	56.65	9.98	29.86	3.503	0.05	0.47	0.1	-0.722
Isosorbide dinitrate	60.27	9.14	28.17	2.417	3.67	-0.37	-1.59	-1.808
Isoxicam	55.09	11.58	30.16	3.171	-1.51	2.07	0.4	-1.054
Isoxsuprine hydrochloride	59.54	9.62	27.35	3.489	2.94	0.11	-2.41	-0.736
Ivermectin	59.49	7.86	29.04	3.548	2.89	-1.65	-0.72	-0.677
Josamycin	53.35	8.77	32.62	5.26	-3.25	-0.74	2.86	1.035
Kanamycin A sulfate	56.65	8.82	29.96	4.563	0.05	-0.69	0.2	0.338
Ketanserin tartrate hydrate	57.38	10.85	28.02	3.669	0.78	1.34	-1.74	-0.556
Ketoconazole	51.94	9.14	33.86	4.965	-4.66	-0.37	4.1	0.74
Ketoprofen	56.15	12.08	29.07	2.701	-0.45	2.57	-0.69	-1.524
Ketorolac tromethamine	59.88	12.44	20.89	6.787	3.28	2.93	-8.87	2.562
Ketotifen fumarate	50.93	7.72	37.65	3.601	-5.67	-1.79	7.89	-0.624
Khellin	60.42	9.82	25.05	4.702	3.82	0.31	-4.71	0.477
L(-)-vesamicol hydrochloride	59.08	8.54	27.77	4.615	2.48	-0.97	-1.99	0.39
Labetalol hydrochloride	47.34	14.93	34.47	3.257	-9.26	5.42	4.71	-0.968
Lanatoside C	64	14.34	20.69	0.966	7.4	4.83	-9.07	-3.259
Lansoprazole	63.89	11.73	19.08	5.074	7.29	2.22	-10.68	0.849
Leflunomide	64.11	6.75	27.86	1.279	7.51	-2.76	-1.9	-2.946
Letrozole	13.98	12.86	67.45	5.705	-42.62	3.35	37.69	1.48
Levocabastine hydrochloride	52.17	12.45	32.62	2.758	-4.43	2.94	2.86	-1.467
Levonordefrin	58.65	10.45	29.28	1.614	2.05	0.94	-0.48	-2.611
Levopropoxyphene napsylate	51.43	16.92	28.39	3.25	-5.17	7.41	-1.37	-0.975
Lidoflazine	54.22	10.18	32.29	3.307	-2.38	0.67	2.53	-0.918
Lincomycin hydrochloride	56.32	9.73	30.17	3.786	-0.28	0.22	0.41	-0.439
Liothyronine	62.64	10.69	25.74	0.936	6.04	1.18	-4.02	-3.289
Lisinopril	49.64	11.48	35.82	3.065	-6.96	1.97	6.06	-1.16
Lithocholic acid	59.23	9.4	29.61	1.762	2.63	-0.11	-0.15	-2.463
Lomefloxacin hydrochloride	59.85	10.33	26.26	3.564	3.25	0.82	-3.5	-0.661
Loracarbef	56.73	12.65	27.05	3.582	0.13	3.14	-2.71	-0.643
Lorglumide sodium salt	61.19	9.03	28.4	1.312	4.59	-0.48	-1.36	-2.913
Lovastatin	60.65	10.69	25.62	3.041	4.05	1.18	-4.14	-1.184
Loxapine succinate	60.82	11.38	23.01	4.79	4.22	1.87	-6.75	0.565
Luteolin	63.23	12.8	23.01	0.968	6.63	3.29	-6.75	-3.257
Lymecycline	48.3	14.97	33.75	2.978	-8.3	5.46	3.99	-1.247
Lynestrenol	56.08	8.87	31.46	3.547	-0.52	-0.64	1.7	-0.678
Mafenide hydrochloride	59.95	9.53	25.02	5.5	3.35	0.02	-4.74	1.275
Maprotiline hydrochloride	34.04	12.25	49.57	4.147	-22.56	2.74	19.81	-0.078
Mebendazole	11.3	9.2	73.43	5.439	-45.3	-0.31	43.67	1.214
Mebeverine hydrochloride	51.51	11.06	33.48	3.951	-5.09	1.55	3.72	-0.274
ebhydroline 1,5-naphtalenedisulfona	53.14	11.27	32.06	3.528	-3.46	1.76	2.3	-0.697
Mecamylamine hydrochloride	51.86	12.32	32.9	2.917	-4.74	2.81	3.14	-1.308
Meclocycline sulfosalicylate	51.35	8.4	35.31	4.934	-5.25	-1.11	5.55	0.709
lofenamic acid sodium salt monohyd	57.88	9.9	28.82	3.339	1.28	0.39	-0.94	-0.886
Meclofenoxate hydrochloride	59.02	8.24	31.55	1.188	2.42	-1.27	1.79	-3.037

Meclozine dihydrochloride	52.26	9.15	34.67	3.833	-4.34	-0.36	4.91	-0.392
Medrysone	67.74	8.61	21.38	2.271	11.14	-0.9	-8.38	-1.954
Mefenamic acid	62.64	14.34	19.29	3.736	6.04	4.83	-10.47	-0.489
Mefexamide hydrochloride	55.8	9.06	31.93	3.094	-0.8	-0.45	2.17	-1.131
Mefloquine hydrochloride	58.24	11.65	25.65	4.471	1.64	2.14	-4.11	0.246
Megestrol acetate	49.19	11.71	33.85	5.249	-7.41	2.2	4.09	1.024
Meglumine	60.68	10.95	26.83	1.542	4.08	1.44	-2.93	-2.683
Melatonin	52.98	11.56	31.3	4.164	-3.62	2.05	1.54	-0.061
Memantine Hydrochloride	56.66	10.96	27.07	5.312	0.06	1.45	-2.69	1.087
Mepenzolate bromide	58.42	10.81	29.62	1.155	1.82	1.3	-0.14	-3.07
Mephenesin	57.84	11.54	27.9	2.716	1.24	2.03	-1.86	-1.509
Mephentermine hemisulfate	61.44	9.22	27.16	2.179	4.84	-0.29	-2.6	-2.046
Mephenytoin	55.11	10.93	29.48	4.48	-1.49	1.42	-0.28	0.255
Meprylcaine hydrochloride	55.65	11.9	29.67	2.786	-0.95	2.39	-0.09	-1.439
Meptazinol hydrochloride	55.19	14	27.86	2.952	-1.41	4.49	-1.9	-1.273
Merbromin	65.14	10.23	22.63	1.997	8.54	0.72	-7.13	-2.228
Meropenem	54.85	11.73	30.04	3.376	-1.75	2.22	0.28	-0.849
Mesalamine	50.8	11.63	33.87	3.706	-5.8	2.12	4.11	-0.519
Mesalamine	51.54	13.56	32.14	2.772	-5.06	4.05	2.38	-1.453
Mesoridazine besylate	55.79	9.11	31.54	3.56	-0.81	-0.4	1.78	-0.665
aproterenol sulfate, orciprenaline sul	51.03	10.93	32.54	5.505	-5.57	1.42	2.78	1.28
Metaraminol bitartrate	59.55	11.86	25.74	2.854	2.95	2.35	-4.02	-1.371
Metergoline	58	9.28	29.12	3.6	1.4	-0.23	-0.64	-0.625
Metformin hydrochloride	56.5	10.57	28.73	4.201	-0.1	1.06	-1.03	-0.024
Methacholine chloride	60.15	8.39	29.89	1.573	3.55	-1.12	0.13	-2.652
Methacycline hydrochloride	56.76	12.83	25.93	4.483	0.16	3.32	-3.83	0.258
Methantheline bromide	57.25	9.92	29.42	3.417	0.65	0.41	-0.34	-0.808
Methapyrilene hydrochloride	60.88	9.54	25.52	4.049	4.28	0.03	-4.24	-0.176
Methazolamide	66.56	11.17	18.94	3.328	9.96	1.66	-10.82	-0.897
Methiazole	15.7	9.39	70.4	3.791	-40.9	-0.12	40.64	-0.434
Methimazole	60.46	9.78	28.32	1.446	3.86	0.27	-1.44	-2.779
Methiothepin maleate	60.57	11.05	24.5	3.884	3.97	1.54	-5.26	-0.341
Methocarbamol	59.85	10.9	24.68	4.571	3.25	1.39	-5.08	0.346
Methotrexate	61.1	9.26	25.9	3.744	4.5	-0.25	-3.86	-0.481
Methotrimeprazine maleat salt	61.26	9.02	28.25	1.467	4.66	-0.49	-1.51	-2.758
Methoxamine hydrochloride	58	9.76	29.53	2.707	1.4	0.25	-0.23	-1.518
Methyl benzethonium chloride	23.73	15.49	59.05	1.733	-32.87	5.98	29.29	-2.492
Methylatropine nitrate	58.97	10.68	29.69	0.667	2.37	1.17	-0.07	-3.558
Methyldopa (L,-)	59.9	10	26.63	3.469	3.3	0.49	-3.13	-0.756
Methyldopate hydrochloride	54.26	12.06	31.29	2.394	-2.34	2.55	1.53	-1.831
Methylergometrine maleate	55.84	9.19	31.93	3.037	-0.76	-0.32	2.17	-1.188
Methylhydantoin-5-(D)	62.71	10.72	24.89	1.594	6.11	1.21	-4.87	-2.631
Methylhydantoin-5-(L)	65.14	7.95	25.91	1.002	8.54	-1.56	-3.85	-3.223
Methylprednisolone, 6-alpha	59.37	8.2	29.22	3.207	2.77	-1.31	-0.54	-1.018
Meticrane	56.37	10.94	28.98	3.711	-0.23	1.43	-0.78	-0.514
Metixene hydrochloride	61.39	9.73	25.25	3.629	4.79	0.22	-4.51	-0.596
Metoclopramide monohydrochloride	59.35	10.8	23.99	5.857	2.75	1.29	-5.77	1.632
Metolazone	54.15	10.8	32.78	2.27	-2.45	1.29	3.02	-1.955
Metoprolol-(+,-) (+)-tartrate salt	59.66	9.81	28.82	1.713	3.06	0.3	-0.94	-2.512

Metrizamide	53.57	10.5	31.42	4.512	-3.03	0.99	1.66	0.287
Metronidazole	60.45	9.47	24.37	5.71	3.85	-0.04	-5.39	1.485
Metyrapone	60.28	10.55	27.33	1.771	3.68	1.04	-2.43	-2.454
Mevalonic-D, L acid lactone	62.54	9.97	26.41	1.08	5.94	0.46	-3.35	-3.145
Mexiletine hydrochloride	55.42	12.46	27.23	4.896	-1.18	2.95	-2.53	0.671
Mianserine hydrochloride	55.15	11.26	29.92	3.663	-1.45	1.75	0.16	-0.562
Miconazole	57.35	9.28	29.58	3.735	0.75	-0.23	-0.18	-0.49
Midodrine hydrochloride	60.37	8.3	29.21	2.118	3.77	-1.21	-0.55	-2.107
Mifepristone	63.35	10.74	23.34	2.573	6.75	1.23	-6.42	-1.652
Milrinone	55.56	11.69	29.8	2.958	-1.04	2.18	0.04	-1.267
Minaprine dihydrochloride	59.99	9.45	27.54	3.025	3.39	-0.06	-2.22	-1.2
Minocycline hydrochloride	54.69	8.69	34.2	2.418	-1.91	-0.82	4.44	-1.807
Mitoxantrone dihydrochloride	69.99	6.2	0.73	23.086	13.39	-3.31	-29.03	18.861
Molindone hydrochloride	50.62	12.5	33.52	3.359	-5.98	2.99	3.76	-0.866
Molsidomine	58.03	11.19	26.43	4.354	1.43	1.68	-3.33	0.129
Mometasone furoate	58.02	10.47	28.47	3.033	1.42	0.96	-1.29	-1.192
Monensin sodium salt	68.12	13.13	16.76	1.993	11.52	3.62	-13	-2.232
Monobenzone	32.84	20.72	44.95	1.488	-23.76	11.2	15.19	-2.737
Morantel tartrate	52.01	11.57	33.52	2.893	-4.59	2.06	3.76	-1.332
Moricizine hydrochloride	56.4	10.27	29.3	3.949	-0.2	0.76	-0.46	-0.276
Moroxidine hydrochloride	56.39	9.69	29.77	4.154	-0.21	0.18	0.01	-0.071
Moxalactam disodium salt	60.93	10.22	28.26	0.582	4.33	0.71	-1.5	-3.643
Moxisylyte hydrochoride	59.94	8.24	28.48	3.344	3.34	-1.27	-1.28	-0.881
Moxonidine	54.61	14.53	29.06	1.796	-1.99	5.02	-0.7	-2.429
N6-methyladenosine	56.33	8.33	32.08	3.265	-0.27	-1.18	2.32	-0.96
Nabumetone	57.9	10.81	29.27	2.016	1.3	1.3	-0.49	-2.209
-Acetyl-DL-homocysteine Thiolacton	61.92	9.78	24.96	3.259	5.32	0.27	-4.8	-0.966
N-Acetyl-L-leucine	53.85	12.17	30.28	3.698	-2.75	2.66	0.52	-0.527
Nadide	57.36	11.34	30.12	1.184	0.76	1.83	0.36	-3.041
Nadolol	61.23	8.57	29.17	1.034	4.63	-0.94	-0.59	-3.191
Nafcillin sodium salt monohydrate	62.22	9.2	27.32	1.252	5.62	-0.31	-2.44	-2.973
Nafronyl oxalate	52.52	11.15	32.71	3.627	-4.08	1.64	2.95	-0.598
Naftifine hydrochloride	60.19	9.71	27.78	2.315	3.59	0.2	-1.98	-1.91
Naftopidil dihydrochloride	51.73	17.7	27.56	3.011	-4.87	8.19	-2.2	-1.214
Nalbuphine hydrochloride	56.01	8.36	32.38	3.25	-0.59	-1.15	2.62	-0.975
Nalidixic acid sodium salt hydrate	56.12	8.6	31.57	3.651	-0.48	-0.91	1.81	-0.574
Naloxone hydrochloride	56.17	8.66	32.7	2.467	-0.43	-0.85	2.94	-1.758
Naltrexone hydrochloride dihydrate	56.19	8.59	31.44	3.789	-0.41	-0.92	1.68	-0.436
Naphazoline hydrochloride	62.79	11.43	21.67	4.112	6.19	1.92	-8.09	-0.113
Naproxen	56.19	10.63	29.79	3.388	-0.41	1.12	0.03	-0.837
Nefopam hydrochloride	54.87	9.06	27.35	8.607	-1.73	-0.45	-2.41	4.382
Neomycin sulfate	57.79	8.14	30.82	3.247	1.19	-1.37	1.06	-0.978
Neostigmine bromide	55.67	12.16	28.65	3.51	-0.93	2.65	-1.11	-0.715
Niacin	60.35	10.54	24.52	4.584	3.75	1.03	-5.24	0.359
Nialamide	48.38	10.08	39.75	1.792	-8.22	0.57	9.99	-2.433
Nicergoline	55.34	8.12	22.7	13.836	-1.26	-1.39	-7.06	9.611
Niclosamide	55.97	9.98	12.62	21.429	-0.63	0.47	-17.14	17.204
Nifenazone	61.67	12.95	12.37	13.01	5.07	3.44	-17.39	8.785
Nifuroxazide	46.15	11.98	37.78	3.992	-10.45	2.47	8.02	-0.233

Nifurtimox	56.19	12.3	28.88	2.625	-0.41	2.79	-0.88	-1.6
Nilutamide	58.58	8.17	31.93	1.32	1.98	-1.34	2.17	-2.905
Nimesulide	57.09	8.37	31.6	2.94	0.49	-1.14	1.84	-1.285
Niridazole	57.21	9.22	30.11	3.467	0.61	-0.29	0.35	-0.758
Nisoxetine hydrochloride	59.26	9.69	29.55	1.497	2.66	0.18	-0.21	-2.728
Nitrocaramiphen hydrochloride	55.27	10.88	30.55	3.307	-1.33	1.37	0.79	-0.918
Nitrofural	53.09	9.64	33.81	3.453	-3.51	0.13	4.05	-0.772
Nitrofurantoin	58.79	9.18	28.61	3.419	2.19	-0.33	-1.15	-0.806
Nizatidine	60.93	10.44	27.72	0.902	4.33	0.93	-2.04	-3.323
Nomegestrol acetate	55.62	10.99	30.89	2.498	-0.98	1.48	1.13	-1.727
Nomifensine maleate	60.65	9.28	26.98	3.092	4.05	-0.23	-2.78	-1.133
Norcyclobenzaprine	60.18	9.08	27.46	3.28	3.58	-0.43	-2.3	-0.945
Norethindrone	59.75	10.13	25.32	4.791	3.15	0.62	-4.44	0.566
Norethynodrel	55.17	13.5	23.84	7.482	-1.43	3.99	-5.92	3.257
Norfloxacin	58.26	13.3	22.13	6.303	1.66	3.79	-7.63	2.078
Norgestrel-(-)-D	64.58	12.42	21.07	1.932	7.98	2.91	-8.69	-2.293
Nortriptyline hydrochloride	59.14	8.12	30.38	2.356	2.54	-1.39	0.62	-1.869
Novobiocin sodium salt	58.14	10.48	30.75	0.626	1.54	0.97	0.99	-3.599
Nystatine	51.51	8.72	36.27	3.504	-5.09	-0.79	6.51	-0.721
Ofloxacin	51.91	10.04	34.48	3.569	-4.69	0.53	4.72	-0.656
Omeprazole	60.65	9.98	26.7	2.662	4.05	0.47	-3.06	-1.563
Ondansetron Hydrochloride	55.09	11.16	31.13	2.608	-1.51	1.65	1.37	-1.617
Ornidazole	59.08	9.2	28.08	3.644	2.48	-0.31	-1.68	-0.581
Oxalamine citrate salt	63.49	9.44	25.65	1.42	6.89	-0.07	-4.11	-2.805
Oxantel pamoate	53.13	10.88	28.8	7.12	-3.47	1.37	-0.96	2.895
Oxaprozin	51.34	14.11	32.05	2.504	-5.26	4.6	2.29	-1.721
Oxethazaine	55.4	13.41	27.59	3.529	-1.2	3.9	-2.17	-0.696
Oxolinic acid	57.14	10.63	27.09	5.067	0.54	1.12	-2.67	0.842
Oxprenolol hydrochloride	51.7	12.99	32.44	2.867	-4.9	3.48	2.68	-1.358
Oxybenzone	52.66	12.33	31.96	2.977	-3.94	2.82	2.2	-1.248
Oxybutynin chloride	56.89	9.16	30.43	3.516	0.29	-0.35	0.67	-0.709
Oxymetazoline hydrochloride	60.23	12.11	14.16	13.501	3.63	2.6	-15.6	9.276
Oxyphenbutazone	49.56	7.16	2.85	40.424	-7.04	-2.35	-26.91	36.199
Oxytetracycline dihydrate	57.46	8.7	30.26	3.584	0.86	-0.81	0.5	-0.641
Ozagrel hydrochloride	49.51	13.19	33.68	3.617	-7.09	3.68	3.92	-0.608
Paclitaxel	13.51	10.38	68.28	6.401	-43.09	0.87	38.52	2.176
Pancuronium bromide	52.01	10.81	34.33	2.841	-4.59	1.3	4.57	-1.384
Panthenol (D)	60.06	9.37	25.43	5.143	3.46	-0.14	-4.33	0.918
Papaverine hydrochloride	61.04	9.97	24.87	4.122	4.44	0.46	-4.89	-0.103
Parbendazole	48	9.82	36.36	5.818	-8.6	0.31	6.6	1.593
Pargyline hydrochloride	53.57	7.88	29.98	8.567	-3.03	-1.63	0.22	4.342
Paromomycin sulfate	58.66	9.23	30.88	1.225	2.06	-0.28	1.12	-3
Parthenolide	49.55	6.77	14.44	29.234	-7.05	-2.74	-15.32	25.009
Pempidine tartrate	60.98	10.47	23.18	5.37	4.38	0.96	-6.58	1.145
Penbutolol sulfate	57.53	10.29	30.04	2.14	0.93	0.78	0.28	-2.085
Pentamidine isethionate	54.36	8.28	34.63	2.731	-2.24	-1.23	4.87	-1.494
Pentetic acid	37.41	25.9	33.81	2.878	-19.19	16.4	4.05	-1.347
Pentolinium bitartrate	56.48	9.5	31.66	2.361	-0.12	-0.01	1.9	-1.864
Pentoxifylline	60.06	10.84	24.57	4.529	3.46	1.33	-5.19	0.304

Pentylenetetrazole	55.87	12.45	27.97	3.72	-0.73	2.94	-1.79	-0.505
Pepstatin A	55.06	8.45	33.22	3.18	-1.54	-1.06	3.46	-1.045
Pergolide mesylate	61.45	10.43	24.86	3.259	4.85	0.92	-4.9	-0.966
Perhexiline maleate	58.26	8.48	29.92	3.337	1.66	-1.03	0.16	-0.888
Perphenazine	53.66	12.76	29.31	4.276	-2.94	3.25	-0.45	0.051
Phenacetin	59.12	11.45	26.2	3.224	2.52	1.94	-3.56	-1.001
Phenazopyridine hydrochloride	61.51	11.13	22.88	4.481	4.91	1.62	-6.88	0.256
Phenelzine sulfate	53.26	11.49	30.76	4.488	-3.34	1.98	1	0.263
Phenethicillin potassium salt	58.09	10.66	26.25	4.876	1.49	1.15	-3.51	0.651
Phenformin hydrochloride	57.31	9.47	30.33	2.898	0.71	-0.04	0.57	-1.327
Phenindione	57.76	9.82	29.36	3.067	1.16	0.31	-0.4	-1.158
Pheniramine maleate	52.37	11	32.18	4.446	-4.23	1.49	2.42	0.221
Phenoxybenzamine hydrochloride	57.12	11.59	29.67	1.535	0.52	2.08	-0.09	-2.69
Phensuximide	58.26	10.44	28.27	3.04	1.66	0.93	-1.49	-1.185
Phentolamine hydrochloride	59.05	12.38	24.1	4.461	2.45	2.87	-5.66	0.236
Phenylpropanolamine hydrochloride	56.15	10.31	29.57	3.978	-0.45	0.8	-0.19	-0.247
Phthalylsulfathiazole	58.99	10.66	29.54	0.813	2.39	1.15	-0.22	-3.412
Picotamide monohydrate	57.16	7.66	32.66	2.517	0.56	-1.85	2.9	-1.708
Picrotoxinin	65.25	7.97	25.76	1.017	8.65	-1.54	-4	-3.208
Pilocarpine nitrate	58.3	9.35	27.8	4.545	1.7	-0.16	-1.96	0.32
Pimethixene maleate	54.96	7.7	34.03	3.307	-1.64	-1.81	4.27	-0.918
Pindolol	54.13	11.57	30.73	3.574	-2.47	2.06	0.97	-0.651
Pipemidic acid	60.59	9.04	29.69	0.683	3.99	-0.47	-0.07	-3.542
Pipenzolate bromide	58.71	10.64	29.6	1.048	2.11	1.13	-0.16	-3.177
Piperacetazine	56.15	11.45	29.42	2.98	-0.45	1.94	-0.34	-1.245
Piperacillin sodium salt	59	10.63	28.98	1.39	2.4	1.12	-0.78	-2.835
Piperidolate hydrochloride	51.85	12.76	32.45	2.943	-4.75	3.25	2.69	-1.282
Piracetam	52.64	11.3	31.14	4.924	-3.96	1.79	1.38	0.699
Pirenperone	58.29	11.3	27.56	2.846	1.69	1.79	-2.2	-1.379
Pirenzepine dihydrochloride	57.37	10.66	28.44	3.529	0.77	1.15	-1.32	-0.696
Piretanide	55.08	10.34	31.36	3.215	-1.52	0.83	1.6	-1.01
Piribedil hydrochloride	54.43	12.13	31.65	1.788	-2.17	2.62	1.89	-2.437
Pirlindole mesylate	57.21	10.41	28.99	3.392	0.61	0.9	-0.77	-0.833
Piromidic acid	56.68	12.07	11.57	19.678	0.08	2.56	-18.19	15.453
Piroxicam	56.21	12.49	27.2	4.094	-0.39	2.98	-2.56	-0.131
Pivampicillin	51.53	11.87	33.08	3.522	-5.07	2.36	3.32	-0.703
Pivmecillinam hydrochloride	55.72	10.02	31.4	2.862	-0.88	0.51	1.64	-1.363
Pizotifen malate	55.84	13.92	28.57	1.668	-0.76	4.41	-1.19	-2.557
Podophyllotoxin	29.13	12.08	54.17	4.44	-27.47	2.57	24.41	0.215
Practolol	57.25	9.72	30.15	2.88	0.65	0.21	0.39	-1.345
Pralidoxime chloride	60.86	10.25	27.68	1.13	4.26	0.74	-2.08	-3.095
Pramoxine hydrochloride	62.3	8.56	27.91	1.234	5.7	-0.95	-1.85	-2.991
Praziquantel	60.02	8	29.19	2.793	3.42	-1.51	-0.57	-1.432
Prazosin hydrochloride	38.32	18.07	41.18	2.353	-18.28	8.56	11.42	-1.872
Prednicarbate	57.97	9.23	29.36	3.448	1.37	-0.28	-0.4	-0.777
Prednisolone	63.87	5.9	26.63	3.606	7.27	-3.61	-3.13	-0.619
Prednisone	54.69	9.43	32.43	3.453	-1.91	-0.08	2.67	-0.772
Pregnenolone	53.59	9.25	29.99	7.174	-3.01	-0.26	0.23	2.949
Prenylamine lactate	58.14	7.71	31.44	2.715	1.54	-1.8	1.68	-1.51

Pridinol methanesulfonate salt	59.71	9.26	29.65	1.392	3.11	-0.25	-0.11	-2.833
Prilocaine hydrochloride	55.39	12.95	27.32	4.203	-1.21	3.44	-2.44	-0.022
Primaquine diphosphate	49.02	10.48	36.86	3.648	-7.58	0.97	7.1	-0.577
Primidone	60.32	9.44	28.54	1.701	3.72	-0.07	-1.22	-2.524
Proadifen hydrochloride	39.7	5.24	2.53	52.53	-16.9	-4.27	-27.23	48.305
Probenecid	53.94	9	33.57	3.489	-2.66	-0.51	3.81	-0.736
Probucol	53.45	13.06	30.55	2.939	-3.15	3.55	0.79	-1.286
Procaine hydrochloride	50.78	14.68	30.99	3.413	-5.82	5.17	1.23	-0.812
Procarbazine hydrochloride	52.94	10.39	32.77	3.896	-3.66	0.88	3.01	-0.329
Prochlorperazine dimaleate	55.16	9.59	32.46	2.705	-1.44	0.08	2.7	-1.52
Procyclidine hydrochloride	50.52	12.15	34.87	2.459	-6.08	2.64	5.11	-1.766
Progesterone	50.59	11.6	30.61	7.197	-6.01	2.09	0.85	2.972
Proglumide	54.83	10.27	32.79	2.114	-1.77	0.76	3.03	-2.111
Proguanil hydrochloride	53.45	12.35	31.75	2.454	-3.15	2.84	1.99	-1.771
Promazine hydrochloride	58.36	10.35	29.93	1.275	1.76	0.84	0.17	-2.95
Promethazine hydrochloride	59.86	9.59	29.08	1.469	3.26	0.08	-0.68	-2.756
Pronethalol hydrochloride	54.26	11.86	30.93	2.868	-2.34	2.35	1.17	-1.357
Propafenone hydrochloride	55.34	8.58	32.92	3.152	-1.26	-0.93	3.16	-1.073
Propantheline bromide	62.05	7.98	28.42	1.547	5.45	-1.53	-1.34	-2.678
Proparacaine hydrochloride	57.04	11.25	30.12	1.594	0.44	1.74	0.36	-2.631
Propidium iodide	59.95	8.9	29.79	1.357	3.35	-0.61	0.03	-2.868
Propofol	59.71	8.4	30.46	1.425	3.11	-1.11	0.7	-2.8
Propoxycaine hydrochloride	54.57	10.94	31.4	3.094	-2.03	1.43	1.64	-1.131
Propylthiouracil	58.29	12.26	25.45	4.006	1.69	2.75	-4.31	-0.219
Proscillaridin A	61.55	17.89	14.72	5.838	4.95	8.38	-15.04	1.613
Protriptyline hydrochloride	59.48	11.05	28.14	1.332	2.88	1.54	-1.62	-2.893
Pyrantel tartrate	57.06	9.07	31.18	2.68	0.46	-0.44	1.42	-1.545
Pyrazinamide	60.86	10.56	25.18	3.397	4.26	1.05	-4.58	-0.828
Pyridoxine hydrochloride	55.35	10.45	31.78	2.418	-1.25	0.94	2.02	-1.807
Pyrilamine maleate	57.58	8.18	31.02	3.162	0.98	-1.33	1.26	-1.063
Pyrimethamine	61.89	6.2	26.8	5.118	5.29	-3.31	-2.96	0.893
Pyrithyldione	62.55	8.21	27.68	1.564	5.95	-1.3	-2.08	-2.661
Pyrvinium pamoate	56.36	10.6	30.68	2.355	-0.24	1.09	0.92	-1.87
Quinacrine dihydrochloride dihydrate	51.66	9.92	35.69	2.722	-4.94	0.41	5.93	-1.503
Quinethazone	53.34	11.3	31.25	4.11	-3.26	1.79	1.49	-0.115
Quinidine hydrochloride monohydrate	56.22	9.09	31.4	3.294	-0.38	-0.42	1.64	-0.931
Quipazine dimaleate salt	56.68	11.55	29.29	2.475	0.08	2.04	-0.47	-1.75
Racecadotril	53.29	9.92	32.9	3.892	-3.31	0.41	3.14	-0.333
Raloxifene hydrochloride	58.87	9.81	30.25	1.063	2.27	0.3	0.49	-3.162
Ramipril	50.68	13.55	32.79	2.981	-5.92	4.04	3.03	-1.244
Ranitidine hydrochloride	54.75	8.45	34.21	2.523	-1.85	-1.06	4.45	-1.702
Remoxipride Hydrochloride	51.31	12.75	32.54	3.405	-5.29	3.24	2.78	-0.82
Repaglinide	61.51	9.86	25.79	2.839	4.91	0.35	-3.97	-1.386
Reserpine	63.26	12.58	15.09	9.07	6.66	3.07	-14.67	4.845
Ribavirin	51.78	13.91	32.07	2.241	-4.82	4.4	2.31	-1.984
Ribostamycin sulfate salt	33.48	10.95	54.57	0.995	-23.12	1.44	24.81	-3.23
Rifabutin	51.08	13.25	32.47	3.203	-5.52	3.74	2.71	-1.022
Rifampicin	55.53	10.4	30.45	3.63	-1.07	0.89	0.69	-0.595
Riluzole hydrochloride	52.7	9.98	33.41	3.906	-3.9	0.47	3.65	-0.319

Rimexolone	53.59	13.8	29.78	2.826	-3.01	4.29	0.02	-1.399
Risperidone	53.43	11.41	32.09	2.978	-3.17	1.9	2.33	-1.247
Ritodrine hydrochloride	56.78	8.83	31.73	2.656	0.18	-0.68	1.97	-1.569
Rolipram	59.02	9.26	29.98	1.741	2.42	-0.25	0.22	-2.484
Ronidazole	48.99	13.97	32.49	4.545	-7.61	4.46	2.73	0.32
Roxithromycin	58.39	9.49	31.02	1.1	1.79	-0.02	1.26	-3.125
S(-)Eticlopride hydrochloride	64	14.34	20.75	0.915	7.4	4.83	-9.01	-3.31
S-(+)-ibuprofen	56.14	11.47	28.56	3.824	-0.46	1.96	-1.2	-0.401
Salbutamol	54.72	11.07	31.15	3.057	-1.88	1.56	1.39	-1.168
Saquinavir mesylate	49.6	9.23	37.48	3.692	-7	-0.28	7.72	-0.533
Scopolamine hydrochloride	59.19	10.23	29.14	1.448	2.59	0.72	-0.62	-2.777
Scopolamin-N-oxide hydrobromide	52.4	9.91	33.68	4.012	-4.2	0.4	3.92	-0.213
Selegiline hydrochloride	52.94	10.45	33.45	3.161	-3.66	0.94	3.69	-1.064
Serotonin hydrochloride	62.2	8.19	26.3	3.309	5.6	-1.32	-3.46	-0.916
Sertaconazole nitrate	55.29	12.08	29.41	3.151	-1.31	2.57	-0.35	-1.074
Simvastatin	62.75	10.99	19.41	6.847	6.15	1.48	-10.35	2.622
Sisomicin sulfate	56.7	9.2	31.39	2.712	0.1	-0.31	1.63	-1.513
Sotalol hydrochloride	57.18	13.18	26.52	3.125	0.58	3.67	-3.24	-1.1
Spectinomycin dihydrochloride	65.15	9.36	21.64	3.855	8.55	-0.15	-8.12	-0.37
Spiperone	57.57	11.77	27.85	2.8	0.97	2.26	-1.91	-1.425
Spiramycin	61.05	9.66	28.36	0.927	4.45	0.15	-1.4	-3.298
Spironolactone	59.4	8.88	27.89	3.836	2.8	-0.63	-1.87	-0.389
Streptomycin sulfate	53.41	8.76	33.4	4.428	-3.19	-0.75	3.64	0.203
Streptozotocin	62.49	8.81	26.93	1.778	5.89	-0.7	-2.83	-2.447
Succinylsulfathiazole	62.8	10.57	23.45	3.1	6.2	1.06	-6.31	-1.125
Sulconazole nitrate	61.29	7.62	29.58	1.506	4.69	-1.89	-0.18	-2.719
Sulfabenzamide	52.06	12.35	33.98	1.533	-4.54	2.84	4.22	-2.692
Sulfacetamide sodic hydrate	59.42	8.6	28.16	3.822	2.82	-0.91	-1.6	-0.403
Sulfachloropyridazine	58.28	10.06	28.73	2.922	1.68	0.55	-1.03	-1.303
Sulfadiazine	59.57	11.5	24.85	4.08	2.97	1.99	-4.91	-0.145
Sulfadimethoxine	61.36	10.31	25.81	2.516	4.76	0.8	-3.95	-1.709
Sulfadoxine	58.64	9.96	27.85	3.547	2.04	0.45	-1.91	-0.678
Sulfaguanidine	60.82	9.07	26.17	3.886	4.22	-0.44	-3.59	-0.339
Sulfamerazine	60.09	10.13	27.2	2.572	3.49	0.62	-2.56	-1.653
Sulfameter	61.15	9.58	28.28	0.996	4.55	0.07	-1.48	-3.229
Sulfamethazine sodium salt	58.79	10.34	29.28	1.585	2.19	0.83	-0.48	-2.64
Sulfamethizole	62.37	9.29	27.19	1.152	5.77	-0.22	-2.57	-3.073
Sulfamethoxazole	55.37	9.49	32.11	3.029	-1.23	-0.02	2.35	-1.196
Sulfamethoxypyridazine	63.32	7.87	27.22	1.591	6.72	-1.64	-2.54	-2.634
Sulfamonomethoxine	59.31	10.48	28.44	1.773	2.71	0.97	-1.32	-2.452
Sulfanilamide	61.31	10.11	27.39	1.194	4.71	0.6	-2.37	-3.031
Sulfaphenazole	56.87	9.44	30.05	3.633	0.27	-0.07	0.29	-0.592
Sulfapyridine	61.4	11.33	23.45	3.824	4.8	1.82	-6.31	-0.401
Sulfaquinoxaline sodium salt	63.27	9.39	25.39	1.959	6.67	-0.12	-4.37	-2.266
Sulfasalazine	53.13	9.79	34.03	3.05	-3.47	0.28	4.27	-1.175
Sulfathiazole	58.57	10.11	28.31	3.014	1.97	0.6	-1.45	-1.211
Sulfinpyrazone	56	8.2	32.16	3.58	-0.6	-1.31	2.4	-0.645
Sulfisoxazole	60.71	11.02	23.28	4.99	4.11	1.51	-6.48	0.765
Sulindac	52.98	10.28	30.68	6.066	-3.62	0.77	0.92	1.841

Sulmazole	58.19	11.38	29.22	1.211	1.59	1.87	-0.54	-3.014
Suloctidil	56.91	8.05	32.28	2.762	0.31	-1.46	2.52	-1.463
Sulpiride	58.3	8.92	30.44	2.346	1.7	-0.59	0.68	-1.879
Suprofen	61.79	10.01	26.84	1.365	5.19	0.5	-2.92	-2.86
Suxibuzone	58.31	12.5	25.76	3.365	1.71	2.99	-4	-0.86
Suxibuzone	58.96	9.87	29	2.164	2.36	0.36	-0.76	-2.061
Tacrine hydrochloride hydrate	54.73	10.28	30.67	4.323	-1.87	0.77	0.91	0.098
Talampicillin hydrochloride	54.43	11.95	30.62	2.927	-2.17	2.44	0.86	-1.298
Tamoxifen citrate	61.45	11.2	23.71	3.636	4.85	1.69	-6.05	-0.589
Telenzepine dihydrochloride	55.22	9.49	32.36	2.925	-1.38	-0.02	2.6	-1.3
Tenoxicam	34.38	9.75	51.52	4.356	-22.22	0.24	21.76	0.131
Terazosin hydrochloride	57.01	10.73	30.8	1.456	0.41	1.22	1.04	-2.769
Terbutaline hemisulfate	49.19	10.2	37.88	2.742	-7.41	0.69	8.12	-1.483
Terconazole	55.35	10.45	31.69	2.508	-1.25	0.94	1.93	-1.717
Terfenadine	60.77	6.89	29.53	2.81	4.17	-2.62	-0.23	-1.415
Testosterone propionate	50.93	12.79	26.67	9.617	-5.67	3.28	-3.09	5.392
Tetracycline hydrochloride	60.14	9.6	26.5	3.763	3.54	0.09	-3.26	-0.462
etrahydroxy-1,4-quinone monohydra	61.97	8.3	28.41	1.322	5.37	-1.21	-1.35	-2.903
Tetrahydrozoline hydrochloride	58.06	11.96	22.06	7.928	1.46	2.45	-7.7	3.703
Thalidomide	58.62	9.59	29.86	1.873	2.02	0.08	0.1	-2.352
Theobromine	61.17	8.76	28.46	1.608	4.57	-0.75	-1.3	-2.617
Theophylline monohydrate	57.39	9.19	32.3	1.117	0.79	-0.32	2.54	-3.108
Thiamine hydrochloride	53.24	9.8	33.99	2.968	-3.36	0.29	4.23	-1.257
Thiamphenicol	56.15	11.46	28.87	3.521	-0.45	1.95	-0.89	-0.704
Thiocolchicoside	55.84	9.03	31.39	3.738	-0.76	-0.48	1.63	-0.487
Thioguanosine	55.07	9.79	31.62	3.522	-1.53	0.28	1.86	-0.703
Thioperamide maleate	60	8.71	29.36	1.774	3.4	-0.8	-0.4	-2.451
Thioproperazine dimesylate	57.59	9.63	29.15	3.63	0.99	0.12	-0.61	-0.595
Thioridazine hydrochloride	58.58	10.13	25.43	5.858	1.98	0.62	-4.33	1.633
Thiorphan	56.91	8.04	31.91	3.147	0.31	-1.47	2.15	-1.078
Thiostrepton	58.07	9.93	29.31	2.69	1.47	0.42	-0.45	-1.535
THIP Hydrochloride	59.85	10.36	25.62	4.161	3.25	0.85	-4.14	-0.064
Thonzonium bromide	59.73	8.09	30.64	1.541	3.13	-1.42	0.88	-2.684
Thyroxine (L)	60.16	10.64	25.28	3.917	3.56	1.13	-4.48	-0.308
Tiabendazole	52.57	9.23	33.9	4.299	-4.03	-0.28	4.14	0.074
Tiapride hydrochloride	59.39	9.06	29.39	2.155	2.79	-0.45	-0.37	-2.07
Tiaprofenic acid	56.75	9.36	30.94	2.951	0.15	-0.15	1.18	-1.274
Ticarcillin sodium	51.37	13.45	32.04	3.14	-5.23	3.94	2.28	-1.085
Ticlopidine hydrochloride	55.38	12.01	28.87	3.734	-1.22	2.5	-0.89	-0.491
Tiletamine hydrochloride	54.97	13.97	28.2	2.862	-1.63	4.46	-1.56	-1.363
Timolol maleate salt	63.37	9.54	25.04	2.05	6.77	0.03	-4.72	-2.175
Tinidazole	60.83	9.73	28.18	1.256	4.23	0.22	-1.58	-2.969
ïratricol, 3,3',5-triiodothyroacetic aci	55.72	8.92	31.51	3.855	-0.88	-0.59	1.75	-0.37
Tobramycin	56.55	9.87	30.99	2.589	-0.05	0.36	1.23	-1.636
Tocainide hydrochloride	55.06	11.83	29.7	3.404	-1.54	2.32	-0.06	-0.821
Todralazine hydrochloride	62.54	11.82	21.18	4.463	5.94	2.31	-8.58	0.238
Tolazoline hydrochloride	60.22	8.44	27.74	3.602	3.62	-1.07	-2.02	-0.623
Tolfenamic acid	61.33	10.76	23.56	4.351	4.73	1.25	-6.2	0.126
Tolmetin sodium salt dihydrate	61.53	9.32	27.71	1.446	4.93	-0.19	-2.05	-2.779

Tolnaftate	58.24	11.08	26.69	3.988	1.64	1.57	-3.07	-0.237
Torsemide	53.6	11.82	32.02	2.568	-3	2.31	2.26	-1.657
Tracazolate hydrochloride	55.34	12.66	29.32	2.672	-1.26	3.15	-0.44	-1.553
Tranexamic acid	61.72	9.86	25.59	2.818	5.12	0.35	-4.17	-1.407
Tranylcypromine hydrochloride	55.82	9.9	28.91	5.311	-0.78	0.39	-0.85	1.086
Trapidil	60.84	10.02	26.31	2.84	4.24	0.51	-3.45	-1.385
Trazodone hydrochloride	57.51	11.68	24.84	5.905	0.91	2.17	-4.92	1.68
Tremorine dihydrochloride	55.54	12.11	28.37	3.979	-1.06	2.6	-1.39	-0.246
Triamcinolone	56.2	11.69	27.96	4.155	-0.4	2.18	-1.8	-0.07
Triamterene	57.84	11.72	26.41	4.025	1.24	2.21	-3.35	-0.2
Tribenoside	48.63	13.28	35.57	2.509	-7.97	3.77	5.81	-1.716
Trichlorfon	60.31	8.19	29.1	2.397	3.71	-1.32	-0.66	-1.828
Trichlormethiazide	58.17	11.44	24.72	5.682	1.57	1.93	-5.04	1.457
Tridihexethyl chloride	58.04	11.82	27.01	3.055	1.44	2.31	-2.75	-1.17
Trifluoperazine dihydrochloride	59.1	7.94	26.22	6.736	2.5	-1.57	-3.54	2.511
Triflupromazine hydrochloride	56.07	8.89	32.32	2.712	-0.53	-0.62	2.56	-1.513
Trifluridine	50.95	12.63	30.32	6.105	-5.65	3.12	0.56	1.88
Triflusal	56.39	10.47	30.09	3.05	-0.21	0.96	0.33	-1.175
Trihexyphenidyl-D,L Hydrochloride	51.38	12.04	35.33	1.249	-5.22	2.53	5.57	-2.976
Trimeprazine tartrate	62.81	9.3	26.79	1.103	6.21	-0.21	-2.97	-3.122
Trimetazidine dihydrochloride	56.25	8.82	31.76	3.168	-0.35	-0.69	2	-1.057
Trimethadione	54.57	10.68	31.35	3.406	-2.03	1.17	1.59	-0.819
Trimethobenzamide hydrochloride	47.08	9.31	30.68	12.934	-9.52	-0.2	0.92	8.709
Trimethoprim	57.93	10.72	27.33	3.946	1.33	1.21	-2.43	-0.279
Trimipramine maleate salt	64.22	10.52	24.22	1.037	7.62	1.01	-5.54	-3.188
Trioxsalen	59.42	8.29	30.84	1.449	2.82	-1.22	1.08	-2.776
Triprolidine hydrochloride	60.19	10.96	24.62	4.237	3.59	1.45	-5.14	0.012
Troleandomycin	58.98	8.36	28.11	4.551	2.38	-1.15	-1.65	0.326
Trolox	56.38	8.34	31.69	3.586	-0.22	-1.17	1.93	-0.639
Tropicamide	59.26	8.64	29.93	2.118	2.66	-0.87	0.17	-2.107
Tyloxapol	58.67	8.92	31.26	1.146	2.07	-0.59	1.5	-3.079
Urapidil hydrochloride	60.63	9.74	27.86	1.771	4.03	0.23	-1.9	-2.454
Urosiol	53.79	11.55	33.98	0.675	-2.81	2.04	4.22	-3.55
Vancomycin hydrochloride	54.37	11.03	31.98	2.629	-2.23	1.52	2.22	-1.596
Verteporfin	49.67	14.37	32.64	3.322	-6.93	4.86	2.88	-0.903
Vidarabine	51.16	11.46	36.71	0.664	-5.44	1.95	6.95	-3.561
Vigabatrin	57.89	11.66	27.16	3.292	1.29	2.15	-2.6	-0.933
Vincamine	59.1	9.13	28.49	3.283	2.5	-0.38	-1.27	-0.942
Vinpocetine	55.27	9.13	32.51	3.099	-1.33	-0.38	2.75	-1.126
Viomycin sulfate	56.46	10.68	30.28	2.585	-0.14	1.17	0.52	-1.64
Xamoterol hemifumarate	63.97	11.19	23.33	1.508	7.37	1.68	-6.43	-2.717
Xylazine	51.89	10.8	34.33	2.981	-4.71	1.29	4.57	-1.244
Xylometazoline hydrochloride	52.71	9.26	34.43	3.538	-3.89	-0.25	4.67	-0.687
Yohimbine hydrochloride	58.25	10.75	26.44	4.556	1.65	1.24	-3.32	0.331
Zalcitabine	45.06	21.23	29.76	3.954	-11.54	11.7	0	-0.271
Zaprinast	52.88	10.81	33.13	3.11	-3.72	1.3	3.37	-1.115
Zardaverine	53.41	11.16	27.89	7.541	-3.19	1.65	-1.87	3.316
Zidovudine, AZT	56.1	9.29	31.37	3.24	-0.5	-0.22	1.61	-0.985
melidine dihydrochloride monohydra	56.45	10.44	27.67	5.443	-0.15	0.93	-2.09	1.218

Zomepirac sodium salt	59.66	8.72	29.98	1.641	3.06	-0.79	0.22	-2.584
Zoxazolamine	59.52	10.57	26.36	3.548	2.92	1.06	-3.4	-0.677

CCI
4.373273831
4.93542055
5.170789978
4.592271878
4.041686406
3.526673929
4.728134516
3.392297304
2.694795911
5.571679819
7.288363397
7.925196591
3.86374171
3.945552433
5.595024665
4.360427043
2.778228932
3.643916163
1.625189527
4.317828274
2.744038812
7.041628789
2.860922229
1.05467578
4.514999557
2.118534635
3.479709183
4.082560471
1.692542466
7.434321489
4.49878039
38.29488681
4.634244814
3.589451351
16.61423862
4.830917201
4.153122199
3.053232549
5.829192826
4.31809275
1.369170917
0.875002286
1.882466733
2.881396189
2.638545812
6.202056111
2.196269565
8.166039432

6.471603279
5.801630461
4.689165064
3.856595001
4.243483946
1.954570029
4.997921968
5.845045851
11.82153801
1.569617788
8.224190477
5.646648209
7.344672151
1.500365289
3.375658306
0.750948733
3.795803604
5.385174928
4.250085176
2.538621673
6.99300429
3.11661098
1.518658948
5.581898333
6.671102158
3.945058175
3.079842366
1.85354687
0.455657766
4.587008175
6.117542399
6.319050641
2.630123381
2.730828629
3.873285556
2.495559256
2.203004539
4.840710795
3.705050607
5.445242052
5.651080339
8.274131314
5.255073739
2.445890635
7.300953705
5.401476557
5.920457837
3.595513315
3.984611022

3.261
6.522678591
6.838738846
6.416007248
7.111902769
2.08780387
13.44666873
4.531770515
3.87060215
7.233665807
4.018191633
7.577135079
3.227096063
2.923222366
9.847150908
4.861185555
4.79532366
2.637187138
5.516738167
24.58227174
10.01141848
3.586436253
2.751785784
4.999774895
2.055699394
30.89644072
9.778560017
2.308787561
2.360583826
3.914644428
3.909419906
3.44205244
5.760410142
6.667290379
5.392399837
2.086185275
5.789272925
13.80827292
2.744690329
2.564156002
4.711146782
4.172587207
1.553199601
3.740719717
3.394764351
9.159811352
5.347271173
6.167951767
1.761141959

5.396413995
5.122996096
5.112193365
2.587527971
6.897544563
4.409288038
3.257014123
3.695007984
2.141078233
6.709444388
4.580196502
4.394820133
5.035159978
4.529387265
1.764903397
6.429032664
5.163245201
1.278630517
5.02153363
37.53023769
2.471441078
1.841445356
5.666913534
48.20678268
8.634289548
4.530792867
2.811911983
2.023739361
4.215365227
3.928853395
4.08253218
1.11977855
5.493321491
4.06319591
4.380991783
15.54449359
2.630204555
1.560456664
4.250886496
6.371482088
6.219834725
2.811487862
5.295806265
5.301464798
4.063899113
6.60594891
3.706335792
3.679298303
19.18703784

9.642835734
4.971991653
3.791661377
3.883150911
3.468547967
3.879470196
19.62776016
8.294064685
0.72861238
2.870208355
6.881959677
2.146074556
8.291327336
39.15155768
4.205826435
3.047434331
5.226042575
7.122157328
4.680318793
6.905868809
3.60950426
2.536351908
27.29175005
2.903463449
4.200095713
1.115019731
1.797871241
5.655908415
4.960650058
5.033275673
3.781830377
8.202142647
95.89742687
4.287751859
1.205767805
2.208913987
6.386171701
6.408363676
7.440647082
2.143961054
3.584737647
6.803346603
4.68888217
8.522120863
2.641027262
4.436368335
4.525930733
5.809984596
1.500167991

6.055370839
3.27982027
5.164574426
2.356001698
4.115971817
2.918687376
15.40189693
2.936583729
3.348396781
9.398999947
11.00378612
10.64485491
2.426728044
2.376489849
2.45517087
4.45799742
8.373184878
6.127702343
3.837681592
0.907705349
8.702445403
5.617118923
2.799910891
2.929044383
3.939668006
4.204202184
4.612543875
5.089874655
4.794162701
5.846505367
7.420213205
3.616579738
2.515057852
3.722051719
0
2.893794049
2.900151893
16.64317965
5.923512556
0.950046841
6.489205575
6.32223228
77.02915682
11.51259814
1.762461063
5.900423375
5.485870578
4.558806423
3.465784327

4.274548046
7.933382696
38.51846478
2.174861145
1.713300908
7.194324152
5.484711114
9.54832467
4.085597753
3.334310274
2.341683369
5.317379524
1.557215785
14.07355069
3.686610367
7.514631129
9.056807881
7.143708351
4.558379537
4.963898569
7.55005298
1.172607778
8.721213734
1.750612464
0.684543644
4.069225479
3.331166312
4.609153935
5.084532329
3.731209589
6.301835368
9.962790573
2.541302029
8.254491444
21.70660185
5.952030242
4.377633607
3.257480008
61.91555863
4.017649064
2.548120091
6.521106118
5.165557182
12.39852205
46.37509219
5.612831371
3.700097972
5.778574997
4.979297139

2.166988925
5.187720983
4.852020198
17.87268799
5.748480582
8.788303363
2.454585301
3.581862086
2.23391674
4.569441979
5.362858286
2.940819784
8.23910341
4.912185257
5.567414122
5.060124109
7.482529051
6.352511393
4.065033825
9.437563245
34.4688323
20.79282013
14.42601178
4.441752019
9.937791706
5.522465482
5.127695486
6.311325138
3.844158686
1.336349131
4.512923221
2.504922354
5.403913767
5.800384815
6.051766354
4.104982826
3.892968148
36.04743221
1.705597842
1.723870355
2.892604536
6.36611687
5.361570759
5.856225832
6.794790946
7.160593551
1.751137059
3.402288788
5.153145059

4.012099326
2.253646157
3.176085012
11.52942622
11.42893648
2.373525858
4.63734148
5.917873351
2.783295888
3.660580419
11.25530648
10.95071614
5.387162983
2.28046048
1.875246384
3.745466059
29.79474855
5.768584662
1.25463142
7.852101375
5.399303288
2.738788783
3.121752873
3.825390046
3.562479614
6.700475804
1.910688096
6.218292049
3.848219848
4.814692514
4.42145406
4.829847306
3.793083706
5.940955226
6.013794476
1.773306516
5.103242989
8.146552891
7.423368575
3.587366165
10.37370927
9.024275262
5.041329587
4.065917363
4.569895404
2.284431001
6.528270904
2.521785082
3.561354939

8.377792609
4.604797064
5.57755762
6.267583266
7.051434251
0.868725503
4.404856865
2.799270619
3.873692812
3.471502413
4.512308168
0.795514928
2.395983306
6.261796867
3.099367032
10.22663405
9.899215929
6.090987523
3.347162978
11.75776867
13.07573635
13.14745987
8.73536582
57.0123969
6.212905037
3.483363461
9.190458367
3.654712574
0.698298647
8.053069042
9.50745497
3.608028409
4.890993866
4.199291488
5.624381655
6.027964499
8.196769181
10.53335412
10.77853
2.007407283
5.942737164
30.14802455
62.93432129
6.49800554
4.565633472
6.475659349
7.752404853
1.859864511
4.460579447

6.574721591
14.10503867
13.02576374
2.613534197
4.921566417
8.804815501
5.873958546
4.436464922
3.244035912
3.800907786
3.375674896
5.869907665
2.092827035
2.948460785
5.232468729
11.36885588
2.964810449
7.436118679
7.055615423
2.103978374
6.512150183
5.680232478
1.677237312
1.481578888
4.572406806
5.077742018
1.165789003
6.02726107
14.82680711
57.65953829
4.976840464
6.776162705
6.198444644
5.953441106
5.642903862
2.092779014
44.5006906
4.432839271
4.636651378
4.203755583
2.607727747
8.332782308
10.02873018
3.273671334
1.723483681
6.609675938
6.721824455
4.539716401
4.080115685

3.605408299
6.78833006
5.1527678
7.523876993
4.116556935
2.346581343
0.942284458
4.518733119
9.540487619
2.727799296
4.226594374
5.224877893
37.26561983
7.719330023
3.996603683
2.454172773
17.88285838
30.47013241
6.420554805
0.95151248
0.285728893
5.896986434
3.896544751
5.962427442
2.787346408
2.915438389
7.235223286
3.896951244
3.647434852
5.731586255
6.817098283
5.32879949
4.527317086
9.854278056
3.079874186
2.159647193
3.555497715
2.006737651
10.36645402
1.457384301
5.310877894
2.317991372
3.103340942
6.535279719
13.17622438
12.07205952
24.29763396
20.42368294
13.40440558

3.359553542
4.343262023
2.56447363
1.073901299
3.820220413
2.261111231
5.416224146
2.622429408
5.900654964
2.72217358
5.046532374
4.379991438
5.508253444
7.976180101
8.924947283
12.36618167
3.501608345
4.15275824
8.332595094
6.707140672
5.331976088
3.081264189
3.066995435
8.499242613
4.81875762
7.552055416
4.673683344
3.063309322
6.719149053
5.465665925
1.076188181
18.69066815
45.71214063
1.43407845
8.918888047
57.84488202
6.749700438
5.619859785
6.621775366
10.96146655
5.544291118
3.817905185
30.28799401
8.044465489
2.428770265
5.699073258
25.59502118
2.664394115
6.385061942

3.554662431
4.069253617
7.022254339
2.15189312
4.405814454
4.877458457
8.610019512
4.01810515
4.035653727
1.609822661
1.715419482
5.100807877
3.441075413
2.691621259
6.806665557
0.969334308
4.327209725
3.881154983
10.17277563
2.619083809
5.004130694
3.421870979
5.356460025
3.980945742
2.373125576
3.957274946
6.481097438
4.612884239
3.577966042
2.035243474
2.565034113
4.59177188
1.571269869
24.00481845
3.950096328
6.541491344
2.362597935
5.289617094
36.8388399
1.558115849
5.709695701
6.764693711
4.043763594
23.26698915
1.648947846
8.721528593
3.373334256
4.228143919
3.273239985

4.215754855
4.387685495
10.4470201
4.658570167
58.12648987
4.732176666
4.980250596
7.922975704
4.83097723
3.417660018
8.553762681
7.074141927
4.165059543
5.008456948
3.540423704
4.323417167
3.769409636
3.686384272
6.403958463
3.205146019
4.452069631
4.385681247
3.183482527
5.389124326
17.98694718
4.654014289
2.192857725
6.396255467
3.129177048
2.333852823
6.96414022
6.964181287
2.364106597
7.87372904
4.008119883
1.969050787
2.729725261
4.592895492
3.934621202
7.880129187
5.219713019
6.84707967
6.473986098
17.10156206
7.201718962
34.09658341
7.269565599
1.663467763
5.37170001

5.424186667
4.543435814
2.614853916
3.483899539
9.238993452
3.815458688
13.04703415
2.377814333
3.043735205
10.43843326
3.914412983
5.762965296
5.387707861
6.775917355
3.097898643
12.41008799
2.24770305
5.051029598
11.79814816
3.424503614
5.715050656
3.447480384
4.90198011
7.012760441
8.980274216
5.744002176
7.330243107
3.391593873
2.42561518
6.075370359
6.46689887
2.910650099
5.568215244
4.674452802
5.772715219
3.561600764
7.027768423
2.909676271
7.825442863
4.004959925
6.111297816
0.71579606
8.144280263
8.290654739
5.633189594
2.794623588
2.872651215
7.85797843
4.234735057

3.924384793
3.273907299
2.689078095
6.62511132
5.348252051
3.244151815
2.219798189
3.617582618
7.957890487
3.217887506
31.10145915
3.22574968
11.11386022
3.019782939
5.12917391
9.029344605
4.835363895
6.367959563
9.007569539
3.103015308
5.475955533
4.103384457
5.553975963
2.300590359
1.924076142
2.524501733
4.285720593
1.311725962
5.071527285
2.835768679
2.212696319
5.331856712
4.448770167
5.840870141
5.784840188
3.52255589
1.749421619
7.017985822
2.9616855
5.180720896
8.534800818
5.408572917
2.078917988
2.07882082
2.90372881
10.69077846
4.326410637
7.898814848
6.022154183

3.82564622
4.710557186
3.757061219
6.760580522
1.631685019
5.662015984
5.706645249
3.142644746
2.856098738
4.205258613
10.69740885
4.391319164
5.806397248
4.042412646
5.248963803
3.083515688
6.745731984
1.56691576
8.575008805
7.561480278
2.390784181
2.947653474
12.93690384
3.035743896
9.996971742
4.279413044
6.435086946
3.132997925
2.355954371
3.50725662
4.045150306
5.091749798
6.51679369
3.841733463
8.986340134
9.714902007
3.730561486
2.982392328
3.277754719
2.085305733
10.28913451
6.802987285
6.121721082
3.923271721
16.4500286
5.303633189
5.233675191
1.964872769
2.59596302

4.088172697
4.654925241

compound names	50uM % cell viability
Hexetidine	0.701838459
Nifuroxazide	6.503458082
Reserpine	0.602349857
Halofantrine	11.41083883
Primaquine	68.18571245
Etoposide	2.234412302
Paclitaxel	1.403489678
Brompheniramine	95.01368605
Saquinavir	44.14310373
Ciclopirox	54.02490872
Parthenolide	0.672623227
Luteolin	5.87206853
Prazosin	2.736372466
Simvastatin	1.520777747
Podophylotoxin	2.345111876
Cyclohexamide	6.250573421
Parbendazole	5.504634181
Nicergoline	59.23318939
GBR 12909	17.69013598
Mitoxantrone	1.029361513
Methyl benzethonium	0.713499977
Fluvastatin	1.748730281
Mebendazole	1.18738005
Norgestrel	1.158077049
Tribenoside	48.52592684
Alexidine	0.584632308
Fenbendazole	1.11715349
Eburnamonine	14.97571736
Colchicine	1.549700417
Estradiol-17 beta	3.287009081
Camptothecine	7.462148341
Methiazole	1.403940224
Niclosalmide	1.269080888
Chlorambucil	65.04795675
Doxorubicin	1.023264523
Digoxin	6.048916351
Diethylstilbestrol	10.80639423
Hycanthone	54.09611829
Medrysone	3.995348266
Monesin	7.556130927
Proscillaridin A	3.936045967
Lanatoside C	6.300578102
Digoxigenin	7.685033703
Doxycycline	25.30108716
Albendazole	85.82199949
Daunorubicin	0.941651453

compound names	IC50 (uM)	Titration Curve
Paclitaxel	0.00324	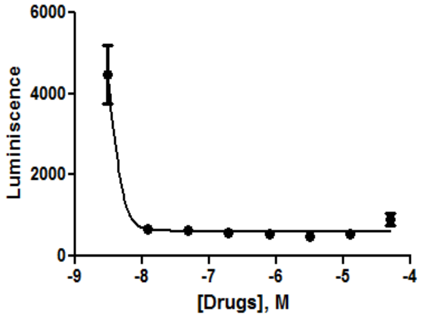
Proscillaridin A	0.005	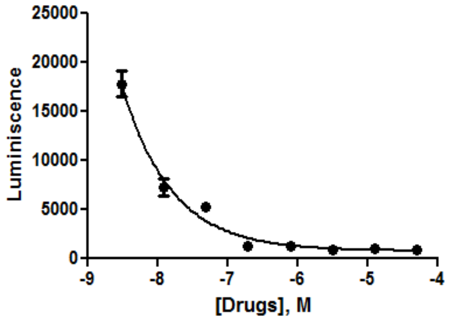
Mitoxantrone	<0.01	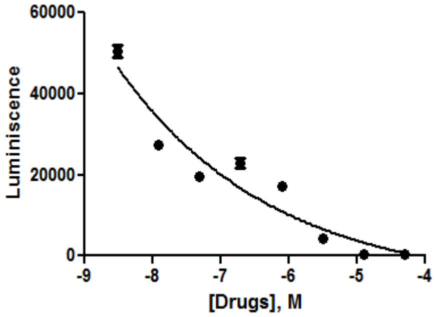
Camptothecin	0.13	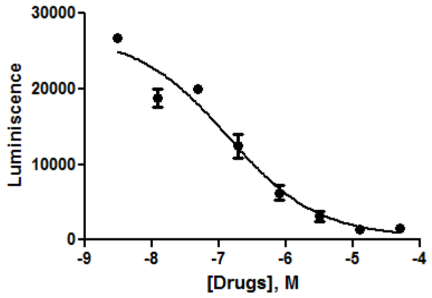

Diethylstilbestrol	0.15	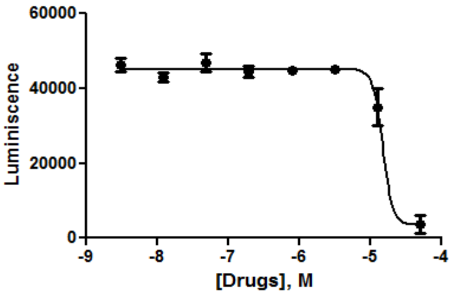
Digoxigenin	0.26	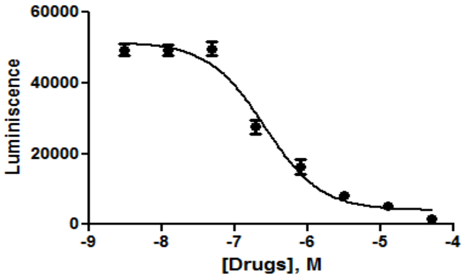
Cyclohexamide	0.33	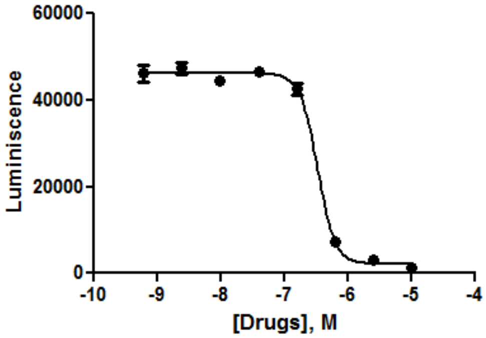
Podophyllotoxin	0.38	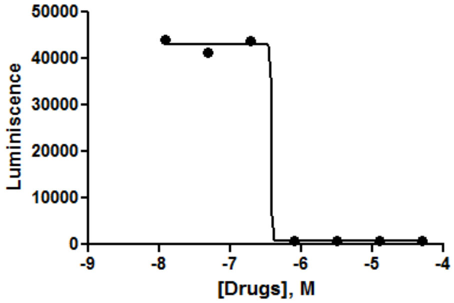

Monensin	0.38	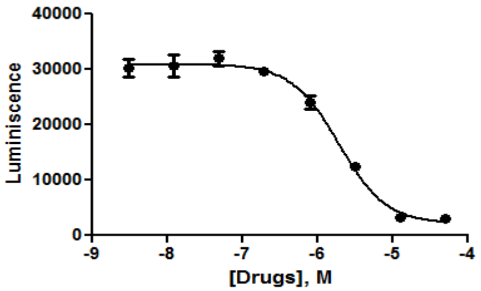
Doxorubicin	0.52	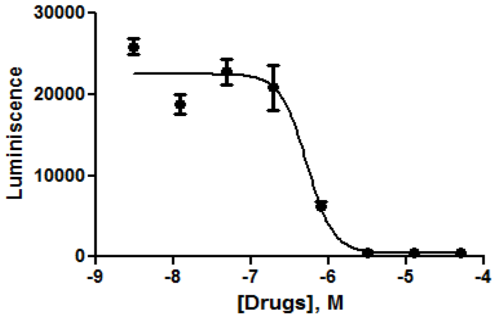
Parbendazole	0.53	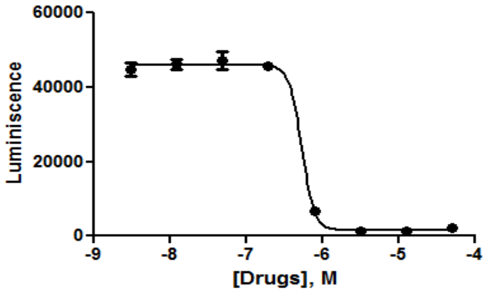
Digoxin	0.73	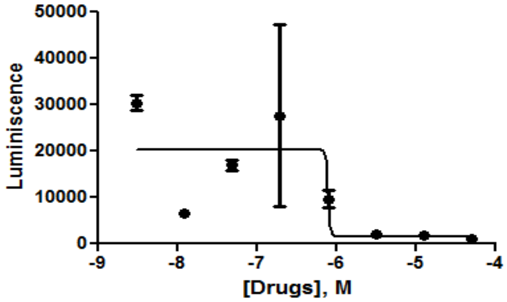

Etoposide	0.92	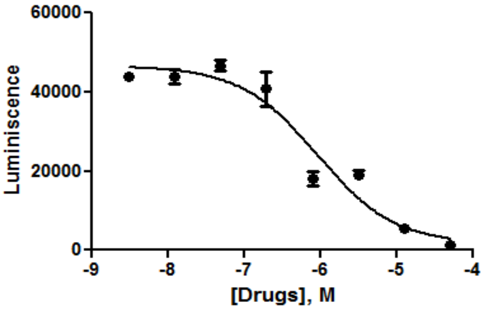
Methiazole	1.37	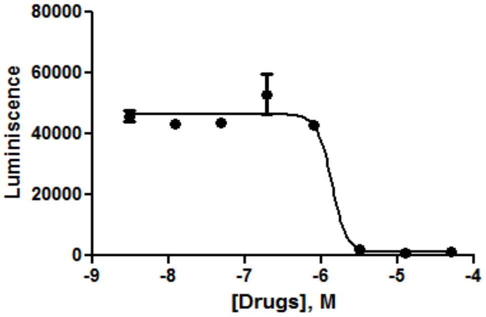
Daunorubicin	1.37	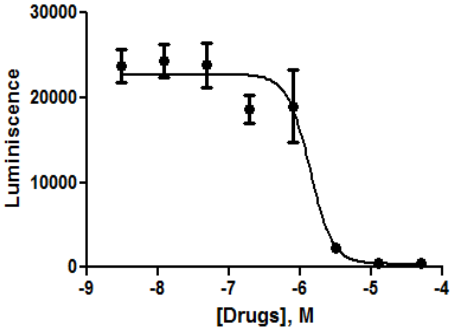
Norgestrel	1.45	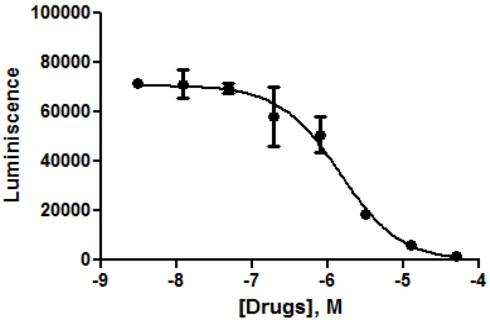

Lanatoside C	1.84	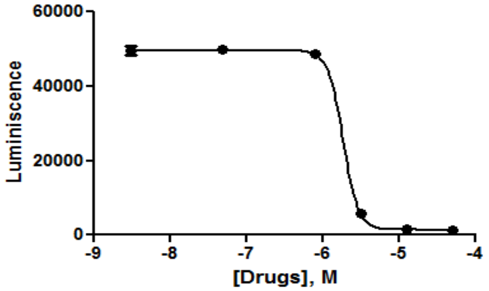
Fenbendazole	2.27	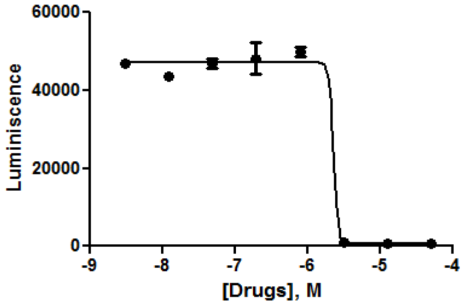
Estradiol-17 beta	2.62	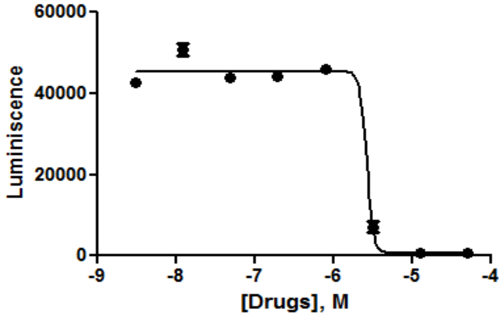
Niclosamide	2.81	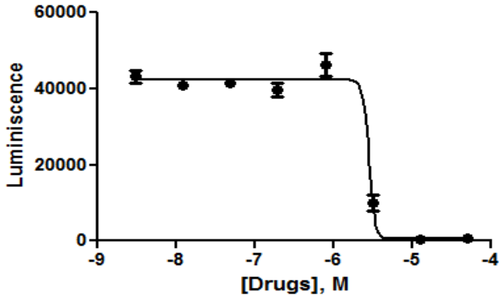

Fluvastatin	3.04	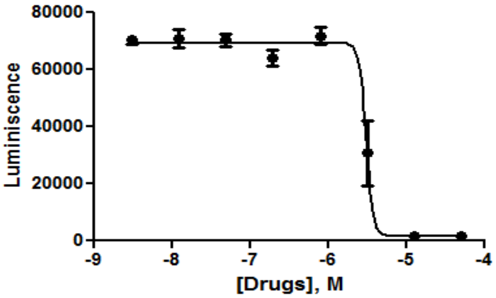
Mebendazole	3.39	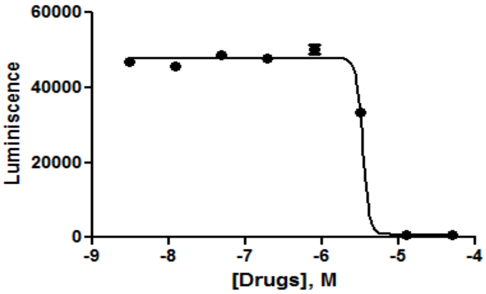
Eburnamonine	8.74	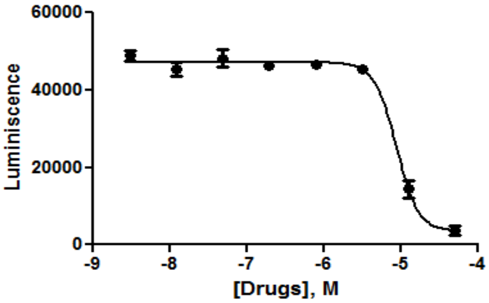
Colchicine	8.79	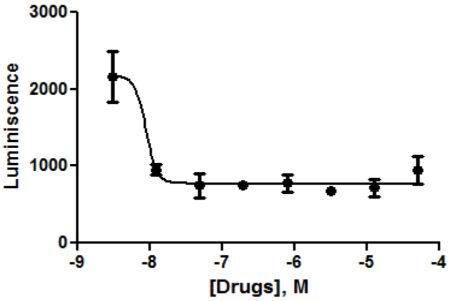

Alexidine	8.81	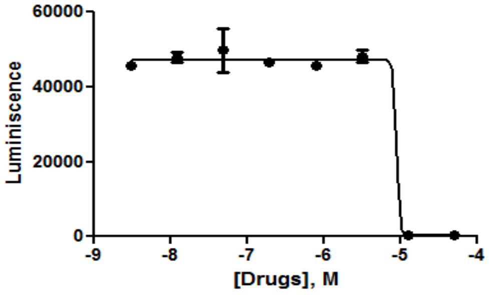
Medrysone	9.13	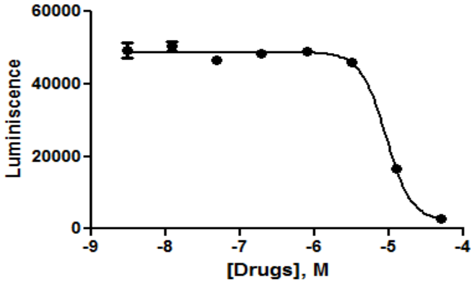
Methyl benzethonium	9.98	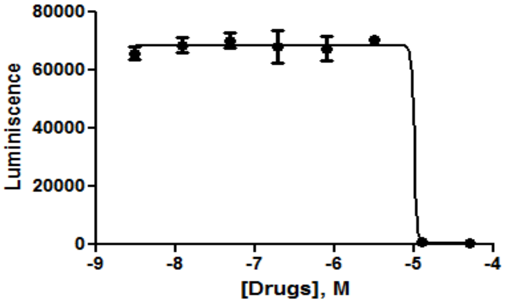
Simvastatin	10.44	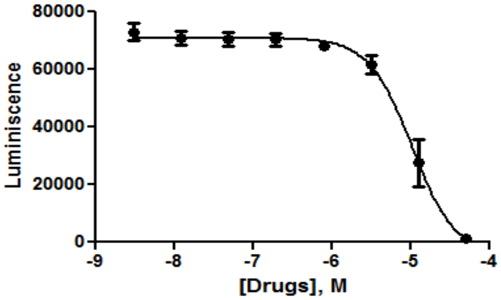

Ciclopirox	11.14	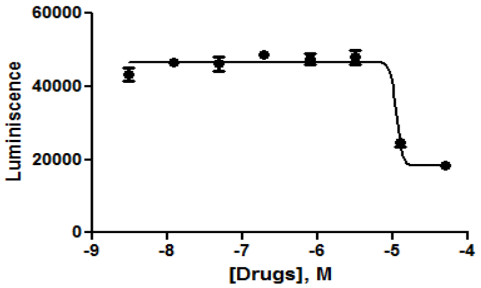
Chlorambucil	12.76	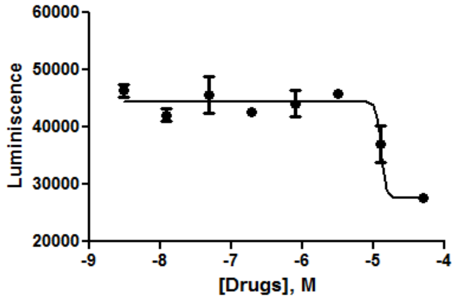
Nicergoline	13.34	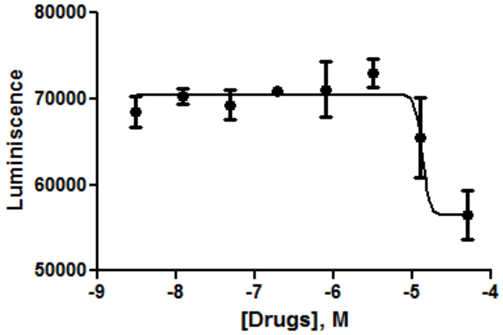
Tribenoside	13.74	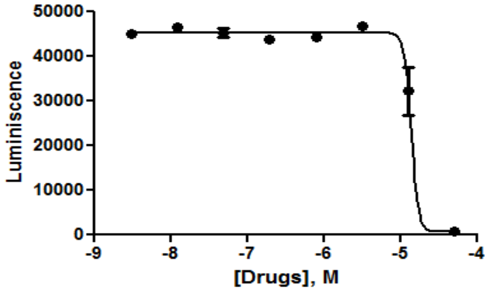

Luteolin	16.1	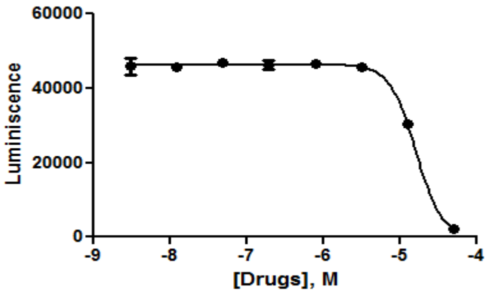
Primaquine	16.16	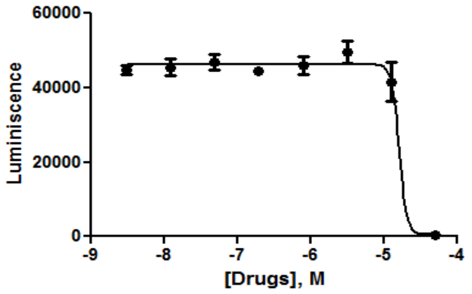
Parthenolide	17.25	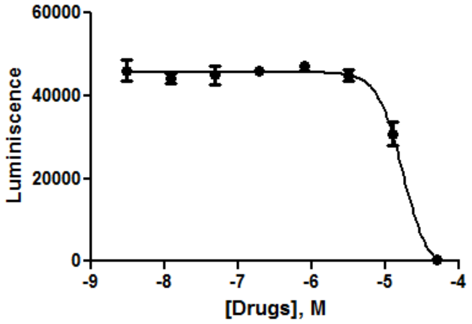
GBR 12909	17.91	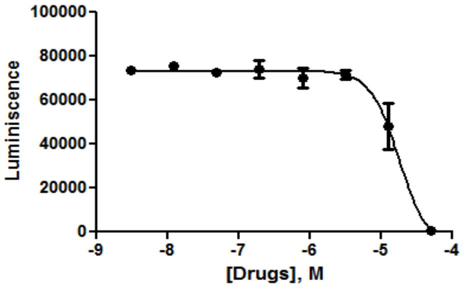

compound names	Fold change/Cell count
Paclitaxel	3.7481473
Camptothecin	0.988664785
Fenbendazole	23.77204166
Niclosamide	0.723619474
Mebendazole	13.19058949
Nicergoline	1.007211985
Mitoxantrone	16.88566977
Doxorubicin	297.302102
Digoxin	0.309420563
Etoposide	8.366031924
Daunorubicin	2773.545522
Estradiol-17 beta	4.077284747
Eburnamonine	8.307548424
Colchicine	18.12612503
Ciclopirox	0.494561746
Primaquine	0.17717033
Parthenolide	0.216485547
GBR 12909	2.912989757
Diethylstilbestrol	3.017339194
Digoxigenin	0.763960007
Cyclohexamide	0.674309218
Podophyllotoxin	13.06552708
Monensin	2.809803672
Norgestrel	4.515228227
Lanatoside C	0.443959821
Fluvastatin	6.582706999
Alexidine	2.151560269
Medrysone	6.940310119
Methyl benzethonium	1.028688726
Simvastatin	3.662120248
Luteolin	8.305231137
Proscillaridin A	0.586440907
Parbendazole	22.50351418
Methiazole	13.09487944
Chlorambucil	5.214370806
Tribenoside	1.881621836
DMSO	2.257272598
